# Multiscale reaction model coupling dual-site microkinetics with bulk diffusion and CFD–DEM for a perovskite oxygen carrier

**DOI:** 10.3389/fchem.2025.1656180

**Published:** 2025-09-04

**Authors:** Ruiwen Wang, Zhenshan Li, Lei Liu

**Affiliations:** 1 Key Laboratory for Thermal Science and Power Engineering of Ministry of Education, Department of Energy and Power Engineering, Tsinghua University, Beijing, China; 2 Hunan Engineering Research Center of Clean and Low-Carbon Energy Technology, School of Energy Science and Engineering, Central South University, Changsha, China

**Keywords:** multiscale model, first-principles calculation, dual-site microkinetics, bulk diffusion, CFD-DEM, oxygen carrier, chemical looping

## Abstract

Non-catalytic heterogeneous reactions in fluidized beds involve physical and chemical processes spanning across the atom, surface, grain, particle, and reactor scales. However, a multiscale modeling framework covering all scales has not been fulfilled due to the incomplete coupling strategies. This study develops a multiscale model coupling all five scales. The elementary reaction path is derived from first-principles calculation, which is applied to a dual-site mean-field microkinetics describing the states of active site pairs; bulk-phase ion diffusion is treated by a lumped parameter method considering the asymmetrical effects of different site types. The intrinsic reaction kinetics is coupled with intraparticle gas diffusion and fluidization computed via CFD–DEM; experimental validation is conducted on a micro-fluidized-bed thermogravimetric analyzer measuring the solid conversion. The model is applied to the reduction of CaMn_0.375_Ti_0.5_Fe_0.125_O_3−*δ*_ by H_2_ at designed gas concentrations and temperatures, revealing the effects of parameters from all scales on the overall reaction kinetics. The developed multiscale framework can be further adopted in other heterogeneous reactions with determined solid microstructures.

## 1 Introduction

Non-catalytic heterogeneous reactions between gases and solid crystals are involved in chemical looping (Ishida and Jin, 1994; Lyngfelt et al., 2001), pollutant removal (Ning et al., 2025; Osman et al., 2021), and other chemical systems (André et al., 2016). The solid materials in such systems, including various types of oxygen carriers (Cho et al., 2004) and adsorbents (Abanades et al., 2005; Lupiáñez et al., 2013), are commonly prepared as porous particles, which is especially applicable to fluidized bed reactors. Multiple physical and chemical processes simultaneously occur in the reactor, which leads to sophisticated impacts of design parameters on the reactor’s performance, requiring to be described by a whole process model.

**FIGURE 1:**
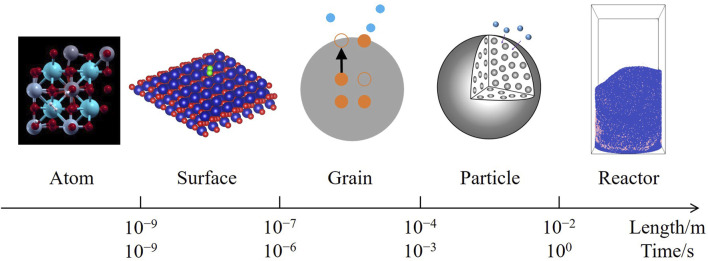
Multiscale modeling framework of non-catalytic heterogeneous reactions.

A multiscale modeling framework has been proposed for catalytic reactions (Bruix et al., 2019), spanning from the microscopic atomic behaviors and surface microkinetics to the macroscopic flows; however, this framework does not cover non-catalytic reactions on the aspect of solid conversion, particularly for solids in internal lattices, compared with catalytic reactions during which the solid components remain unchanged. A complete framework for non-catalytic heterogeneous reactions can be constructed by integrating mesoscale mass transfer processes between microkinetics and fluidization, which consists of five scales as Figure 1, including:

1. The atom scale, where solid atoms and gas molecules interact, triggering an elementary reaction;
2. The surface scale, where reaction intermediates are adsorbed onto active sites distributed on the solid surface, according to Langmuir’s adsorption model (Langmuir, 1916);
3. The grain scale, where solid ions migrate between the surface and internal lattices of a grain, converting the solid reactant to the product;
4. The particle scale, where gas molecules diffuse through pores in a particle, before reaching the inner grain surfaces;
5. The reactor scale, where the particles are fluidized by the gas, forming a two-phase flow.

Various validated models for every independent scale are found in the literature. At the atom scale, the reaction paths and rate constants are directly calculated from the material properties with DFT (density functional theory), also known as the first-principles calculation (Bhandari et al., 2020; Fang et al., 2022; Li et al., 2023; Liu T. et al., 2021; Noor et al., 2020; Wang X. et al., 2024; Yan et al., 2020), which is a rigorous theoretical model requiring no empirical parameters. At the surface scale, the microkinetics, evolving the coverages of different species on the active sites, is modeled with mean-field approximation (Madon et al., 2011; Maestri et al., 2008; Thybaut et al., 2011) or kinetic Monte Carlo (Andersen et al., 2019; Reuter, 2016); elementary reactions may occur by single-site mechanisms including adsorption–desorption and Eley–Rideal, or multi-site mechanisms such as dissociation–association and Langmuir–Hinshelwood (Motagamwala and Dumesic, 2021); dual-site mechanisms are possible on a surface with different types of active sites (Van Belleghem et al., 2022; D’Ambrosio et al., 2024). At the grain scale, the solid conversion process can be described by homogeneous bulk diffusion (Arangio et al., 2015; Stearn and Eyring, 1940; Willis and Wilson, 2022), shrinking core model (Ishida and Wen, 1971; Szekely and Evans, 1970), or a more complex product island model (Fang et al., 2011; Li, 2020), based on the structure of the solid reactant. At the particle scale, intraparticle gas diffusion is controlled by the distribution of grains and pores, shaped by either grain models (Dam-Johansen et al., 1991; Gibson III and Harrison, 1980; Szekely and Evans, 1971) or pore models (Bhatia and Perlmutter, 1980; Bhatia and Perlmutter, 1981; He et al., 2013; Petersen, 1957; Sandmann Jr and Zygourakis, 1986). At the reactor scale, numerical simulation is applied to the two-phase flow to predict the fluidization behavior, the gas phase evolved with CFD (computational fluid dynamics), and the dense particle phase modeled with TFM (two-fluid model) (Ishii and Mishima, 1984), MP-PIC (multiphase particle-in-cell) (Andrews and O'Rourke, 1996) or DEM (discrete element method) (Cundall and Strack, 1979; Golshan et al., 2020); as the computational capacity develops, the most detailed and computationally expensive CFD–DEM model has been increasingly adopted, supporting up to 10^5^–10^8^ particles (Golshan et al., 2020).

Despite the variety of abovementioned models, the coupling between adjacent scales, which multiplies the computational costs of both models, has been an obstacle to completing the framework. Existing studies have proposed several coupling strategies for particular scales by simplifying the larger scale to identical sub-processes of the smaller scale. Microscopic coupling, suggested by studies on catalytic microkinetics (Alexopoulos et al., 2016; Chen and Wang, 2024; Jørgensen and Grönbeck, 2016; Rawal et al., 2021; Yin et al., 2020; Yu et al., 2023), assumes that active sites uniformly distribute on the surface, all sites sharing the same reaction paths derived from first-principles calculation; on this basis, the difference among sites is omitted in single-site reactions, while in multi-site reactions, the state of every pair of neighboring sites shall be considered (Razdan and Bhan, 2021). Macroscopic coupling appears in both CFD–DEM studies numerically solving intraparticle diffusion (Hadian et al., 2024), and particle-scale models analytically expressing the reaction rate with the surface gas concentration (Sedghkerdar and Mahinpey, 2015; Wang et al., 2017; Wang et al., 2021; Yang et al., 2016). The above micro- and macroscopic coupling strategies have been generally agreed in the literature; in contrast, mesoscopic coupling, focusing on the relation between microkinetics and solid conversion, has not undergone sufficient research. Some studies have introduced a bulk-phase ion diffusion process, associating the surface coverage of active ions with its bulk concentration (Li, 2022; Li L. et al., 2021; Liu et al., 2022; Wang et al., 2025; Wang Y. et al., 2024); others have described a phase transformation process based on the shrinking core model, which is integrated into the microkinetics (Cai and Li, 2024a; Cai and Li, 2024b; Cai and Li, 2024c).

However, none of above coupling models have accomplished a complete multiscale framework as Figure 1. Studies on microkinetics have not considered the solid conversion processes of a real porous particle; CFD–DEM studies, mainly applied to the combustion of organic solid fuels, whose molecular structures are unclear, have not developed verifiable microkinetics. Consequently, parameters from the absent scales rely on experimental fitting, instead of theoretical calculation or direct measurement. Models with mesoscopic coupling, excluding the reactor-scale, are unable to provide adequate information of the diverse particles; furthermore, only surfaces with a single type of active sites are involved in these studies (Cai and Li, 2024a; Cai and Li, 2024b; Cai and Li, 2024c; Li, 2022; Li T. et al., 2021; Liu et al., 2022; Wang et al., 2025; Wang Y. et al., 2024), while dual-site reaction mechanisms have not been discussed, resulting in a limited scope of application.

The aim of this study is to develop a multiscale model for non-catalytic heterogeneous reactions under the framework of Figure 1, coupling strategies introduced between every two adjacent scales. The atom-scale reaction path is derived from first-principles calculation; the surface-scale mean-field microkinetics describes the coverages of two types of active sites; the grain-scale ion diffusion is coupled with the microkinetics by the lumped parameter method; the particle-scale gas diffusion is treated under a uniform grain model; the reactor-scale fluidization is simulated with CFD–DEM. Experimental validation is conducted on a micro-fluidized-bed thermogravimetric analyzer (MFB–TGA) (Li et al., 2019). Without loss of generality, the dual-site reaction of a perovskite oxygen carrier (CaMn_0.375_Ti_0.5_Fe_0.125_O_3−*δ*_, CMTF8341) reduced by H_2_ is considered. Kinetics of this reaction has been experimentally studied in a prior work (Liu L. et al., 2021), where the material is prepared by spray drying and calcination, sieved between 180 and 250 μm, and tested on MFB–TGA; fast reaction kinetics and good stability have been observed under H_2_/O_2_ redox cycles. The gas reactant, H_2_, offers a special dual-site mechanism as needed, while fewer elementary reactions are involved compared with other reducing agents. Moreover, the perovskite structure allows oxygen anions to undergo homogeneous bulk diffusion, rather than changing the surface structure into another phase. Thus, this reaction is expected to concisely and clearly describe the full modeling framework.

## 2 Model

The overall reaction of H_2_ reducing CMTF8341 as Equation 1.

$$H_{2}+\frac{1}{\delta }\text{Ca}{\text{Mn}}_{0.375}{\text{Ti}}_{0.5}{\text{Fe}}_{0.125}O_{3}\to H_{2}O+\frac{1}{\delta }\text{Ca}{\text{Mn}}_{0.375}{\text{Ti}}_{0.5}{\text{Fe}}_{0.125}O_{3-\delta }$$ (1)

Complete reduction is achieved when δ=0.5 , corresponding to a solid mass loss of 5.73%; however, the actual mass loss, as measured in Section 3.1, is less than the theoretical value due to the impurity in CMTF8341. Given that the solid mass decreases from $m_{\text{ox}}$ to a limit of $m_{\text{re}}$ , when the reactive component is completely reduced, the mass capacity of the oxygen carrier material is defined as Equation 2.

$$R_{\text{OC}}=\frac{m_{\text{ox}}-m_{\text{re}}}{m_{\text{ox}}}$$ (2)

The solid conversion is defined by the real-time solid mass, $m\left(t\right)$ , as Equation 3.

$$X=\frac{m_{\text{ox}}-m\left(t\right)}{m_{\text{ox}}R_{\text{OC}}}$$ (3)

which is applicable to a grain, a particle, or all CMTF8341 particles in a reactor. The conversion increases from 0 to 1 during the whole reaction process. The final output of the model is the change of conversion against time in a reactor, which is also measured by the experiment as Section 3.

### 2.1 Atom scale

**FIGURE 2:**
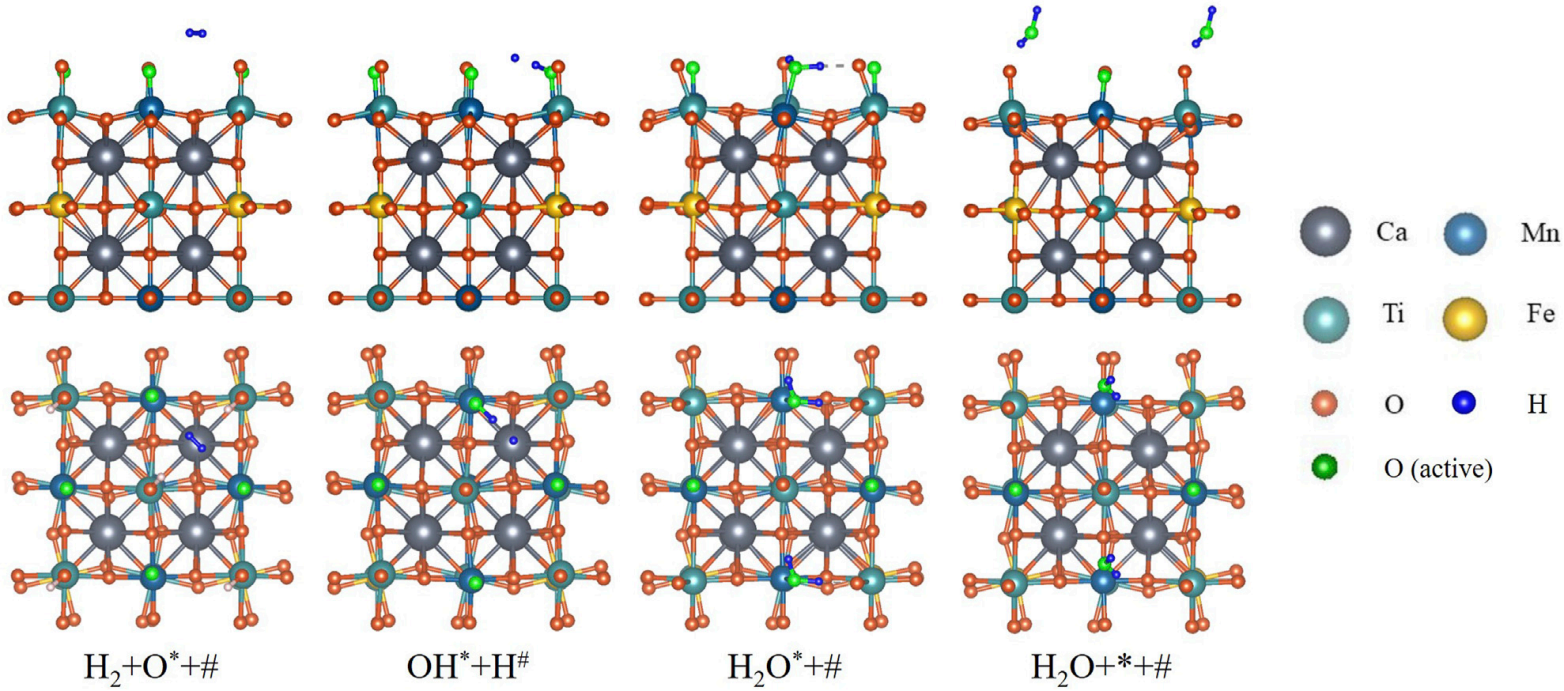
Reaction path of CMTF8341 reduced by H_2_ from DFT calculation. The H_2_ molecule undergoes asymmetrical dissociation, re-association, and desorption to become H_2_O.

DFT calculation is conducted for H_2_ reducing fully oxidized CMTF8341. The reaction path is illustrated in Figure 2, consisting of (three elementary steps) as Equations 4–6. First, one H_2_ molecule undergoes a dissociative adsorption onto the surface; one H atom is combined with an active O atom on a Mn site, forming a hydroxyl (OH) radical; the other H atom is more likely to be placed on a Ca atom, rather than another Mn site. With Mn sites represented by * signs, and Ca sites by # signs, the elementary reaction is expressed as

$$H_{2}\left(g\right)+O^{*}+\#⇌{\text{OH}}^{*}+H^{\#}$$ (4)

Subsequently, the single H atom is associated with the OH radical, forming one H_2_O molecule on the Mn site.

$${\text{OH}}^{*}+H^{\#}⇌{H_{2}O}^{*}+\#$$ (5)

The last step is the H_2_O molecule desorbing into the gas phase, leaving a vacant Mn site on the surface.

$${H_{2}O}^{*}⇌H_{2}O\left(g\right)+*$$ (6)

**FIGURE 3:**
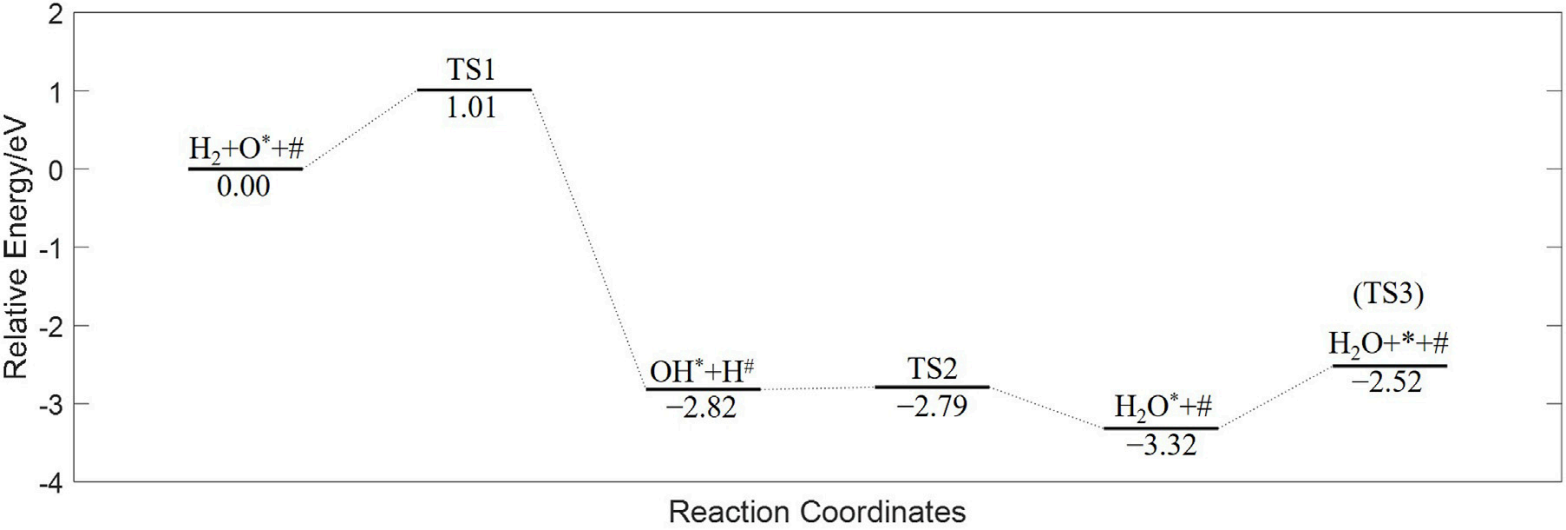
Energy diagram of CMTF8341 reduced by H_2_ from DFT calculation.

The energy diagram, having undergone zero-point correction, is plotted in Figure 3. The vibration frequencies of the species and transition states, along with other basic parameters, are listed in Table 1.

**TABLE 1:** Properties of species and transition states involved in the reaction path.

Parameter	$H_{2}$	$H_{2}O$	$O^{*}$	${\text{OH}}^{*}$	${H_{2}O}^{*}$	$H^{\#}$	TS1	TS2	TS3
Translation type	3D	3D	Fixed	Fixed	Fixed	Fixed	Fixed	Fixed	2D
Molar mass/(g⋅mol^−1^)	2	18	—	—	—	—	—	—	18
Rotation type	Linear	Non-linear	Fixed	Fixed	Fixed	Fixed	Fixed	Fixed	Non-linear
Moment of inertia/(kg⋅m^2^)	4.56 × 10^−48^	1.01 × 10^−47^ 1.90 × 10^−47^ 2.92 × 10^−47^	—	—	—	—	—	—	1.01 × 10^−47^ 1.90 × 10^−47^ 2.92 × 10^−47^
Symmetry number	2	2	—	—	—	—	—	—	2
Vibration frequencies/THz	128.11	103.99 100.39 49.08	—	108.57 26.51 16.80 9.37 6.08	109.84 79.50 47.84	—	75.14 28.14 25.97 17.70 16.67 12.43 7.27 6.83	109.25 42.32 32.90 26.18 21.88 16.87 15.07 7.51	103.99 100.39 49.08

### 2.2 Surface scale

#### 2.2.1 Mean-field assumption

The surface of the CMTF8341 crystal is highly periodical, where both Mn sites and Ca sites are evenly distributed. A mean-field assumption is employed in treating the elementary reactions on different sites, which neglects the change of the surface force field caused by different adsorbates, so that all sites are in an identical environment. Consequently, elementary reactions on every site occur along the same paths as Figure 2, while every site shares an equal reacting probability.

Previous studies on single-site reactions, based on the mean-field assumption, have treated the surface species as non-localized independent particle systems (Cai and Li, 2024a; Cai and Li, 2024b; Cai and Li, 2024c; Liu et al., 2022; Wang et al., 2025). A dual-site reaction, however, can only occur on an adjacent pair of sites, rather than every pair; thus, the surface species shall not be treated as completely independent for dual-site reactions. Alternatively, a pair of sites can be regarded independent of other pairs, given that one elementary reaction does not interfere with reactions on other sites.

Consider a CMTF8341 surface, where the numbers of Mn sites and Ca sites are $N_{*,\text{tot}}$ and $N_{\#,\text{tot}}$ , respectively. Every Mn site has an equal number of neighboring Ca sites, the number denoted as $d_{*}$ , while every Ca site has $d_{\#}$ neighboring Mn sites. The total number of Mn–Ca site pairs on the surface is thus as Equation 7.

$$N_{*\#,\text{tot}}=N_{*,\text{tot}}d_{*}=N_{\#,\text{tot}}d_{\#}$$ (7)

Assuming that a Mn site is occupied by a surface species $X^{*}$ , and its neighboring Ca site by $Y^{\#}$ , the state of this site pair can be expressed as $X^{*}Y^{\#}$ . As $X^{*}$ and $Y^{\#}$ randomly distribute on their corresponding sites, the number of $X^{*}Y^{\#}$ pairs has a mathematical expectation of Equation 8

$$N_{X^{*}Y^{\#}}=N_{*\#,\text{tot}}\theta_{X^{*}}\vartheta_{Y^{\#}}$$ (8)

where $\theta_{X^{*}}$ is the coverage of $X^{*}$ on Mn sites, and $\vartheta_{Y^{\#}}$ that of $Y^{\#}$ on Ca sites.

#### 2.2.2 Rate equation

The rate of an elementary reaction is derived from the transition state theory. The transition state of the single-site reaction (Equation 6) is formed on a Mn site, so the rate equation is based on the Mn-site coverages, as Equation 9.

$$\left\{\begin{array}{l} {\dot{\theta }}_{\text{ER}3}=\frac{{\dot{N}}_{\text{ER}3}}{N_{*,\text{tot}}}=k_{+3}\theta_{{H_{2}O}^{*}}-k_{-3}p_{H_{2}O}\theta_{*}\\ k_{+3}=\frac{k_{B}T}{h}\frac{q_{\text{TS}3}^{0,‡}}{q_{{H_{2}O}^{*}}^{0}}\exp\left(-\frac{\Delta \varepsilon_{0,+3}}{k_{B}T}\right)\\ k_{-3}=\frac{1}{h}\frac{q_{\text{TS}3}^{0,‡}}{\frac{q_{H_{2}O}^{0}}{V}q_{*}^{0}}\exp\left(-\frac{\Delta \varepsilon_{0,-3}}{k_{B}T}\right) \end{array}\right.$$ (9)

where every $q^{0}$ represents the partition function of a species (or transition state) derived from statistical mechanics; the energy barriers, $\Delta \varepsilon_{0}$ , are given by Figure 3.

As for a dual-site reaction, taking Equation 4 for example, the transition state is formed on a site pair, so the rate is expressed on a site-pair basis, as Equation 10

$$\frac{{\dot{N}}_{\text{ER}1}}{N_{*\#,\text{tot}}}=k_{+1}p_{H_{2}}\theta_{O^{*}}\vartheta_{\#}-k_{-1}\theta_{{\text{OH}}^{*}}\vartheta_{H^{\#}}$$ (10)

where ${\dot{N}}_{\text{ER}1}$ is the net turnover frequency of Equation 4 on the entire surface. Whereas the solid reactant and product species ( $O^{*}$ and * ) are located on Mn sites, the rate equation is further expressed with the Mn-site coverage. Given Equation 11

$${\dot{\theta }}_{\text{ER}1}=\frac{{\dot{N}}_{\text{ER}1}}{N_{*,\text{tot}}}$$ (11)

the rate equation is transformed into Equation 12.

$$\left\{\begin{array}{l} {\dot{\theta }}_{\text{ER}1}=d_{*}k_{+1}p_{H_{2}}\theta_{O^{*}}\vartheta_{\#}-d_{*}k_{-1}\theta_{{\text{OH}}^{*}}\vartheta_{H^{\#}}\\ k_{+1}=\frac{1}{h}\frac{q_{\text{TS}1}^{0,‡}}{\frac{q_{H_{2}}^{0}}{V}q_{O^{*}}^{0}q_{\#}^{0}}\exp\left(-\frac{\Delta \varepsilon_{0,+1}}{k_{B}T}\right)\\ k_{-1}=\frac{k_{B}T}{h}\frac{q_{\text{TS}1}^{0,‡}}{q_{{\text{OH}}^{*}}^{0}q_{H^{\#}}^{0}}\exp\left(-\frac{\Delta \varepsilon_{0,-1}}{k_{B}T}\right) \end{array}\right.$$ (12)

The above derivation is the same for Equation 5, resulting in Equation 13.

$$\left\{\begin{array}{l} {\dot{\theta }}_{\text{ER}2}=d_{*}k_{+2}\theta_{{\text{OH}}^{*}}\vartheta_{H^{\#}}-d_{*}k_{-2}\theta_{{H_{2}O}^{*}}\vartheta_{\#}\\ k_{+2}=\frac{k_{B}T}{h}\frac{q_{\text{TS}2}^{0,‡}}{q_{{\text{OH}}^{*}}^{0}q_{H^{\#}}^{0}}\exp\left(-\frac{\Delta \varepsilon_{0,+2}}{k_{B}T}\right)\\ k_{-2}=\frac{k_{B}T}{h}\frac{q_{\text{TS}2}^{0,‡}}{q_{{H_{2}O}^{*}}^{0}q_{\#}^{0}}\exp\left(-\frac{\Delta \varepsilon_{0,-2}}{k_{B}T}\right) \end{array}\right.$$ (13)

In brief, the rate constants of dual-site reactions shall be multiplied by the number of neighbors ( $d_{*}$ ) when considering one type of sites. This result corresponds to the theory by Nørskov et al. (2014), which suggests that every neighbor provides an equivalent reaction path, thus increasing the reacting probability by a factor of $d_{*}$ .

### 2.3 Grain scale

#### 2.3.1 Bulk ion diffusion

The reduction of CMTF8341 is achieved by the removal of oxygen anions ( $O^{2-}$ ) in the lattices, while only surface oxygen ( $O^{*}$ ) is consumed through Equation 4. Lattice oxygen does not directly participate in the surface reactions; instead, it diffuses outward from the internal lattices to the grain surface, driven by its concentration gradient. The structure of CMTF8341 remains stable during the process, forming a solid solution where lattice oxygen ( $O_{O}$ ) and vacancies ( $V_{O}$ ) act as crystal defects, rather than mixed phases of crystals.

The ion diffusion process is fast enough in perovskites (Li, 2022), so that the concentration gradient is negligible. A lumped parameter method is employed by treating $O_{O}$ and $V_{O}$ as two non-localized independent particle systems. The surface–lattice oxygen transformation can be expressed as

$$O_{O}+*⇌V_{O}+O^{*}$$ (14)

The equilibrium of Equation 14 is reached by equalizing the surface and lattice oxygen concentrations, given by Equation 15

$$\frac{\theta_{O^{*}}}{\theta_{*}}=\frac{C_{O_{O}}}{C_{V_{O}}}$$ (15)

where C is the concentration of $O_{O}$ or $V_{O}$ . The diffusion rate is expressed as Equation 16

$${\dot{\theta }}_{\text{ER}4}=\frac{J}{\Lambda }$$ (16)

where J is the surface diffusion flux, and Λ the areal Mn-site density.

The overall reaction is the sum of elementary reactions Equations 4, 5, 6, 14, as Equation 17

$$H_{2}\left(g\right)+O_{O}⇌H_{2}O\left(g\right)+V_{O}$$ (17)

which is equivalent to Equation 1.

#### 2.3.2 Non-catalytic microkinetics

Given the rates of surface reactions Equations 4–6 and ion diffusion Equation 14, the complete microkinetics on Mn sites is expressed as Equation 18

$$\left\{\begin{array}{l} \frac{d\theta_{O^{*}}}{dt}=-{\dot{\theta }}_{\text{ER}1}+{\dot{\theta }}_{\text{ER}4}\\ \frac{d\theta_{{\text{OH}}^{*}}}{dt}={\dot{\theta }}_{\text{ER}1}-{\dot{\theta }}_{\text{ER}2}\\ \frac{d\theta_{{H_{2}O}^{*}}}{dt}={\dot{\theta }}_{\text{ER}2}-{\dot{\theta }}_{\text{ER}3}\\ \frac{d\theta_{*}}{dt}={\dot{\theta }}_{\text{ER}3}-{\dot{\theta }}_{\text{ER}4} \end{array}\right.$$ (18)

while on Ca sites, there is Equation 19.

$$\left\{\begin{array}{l} \frac{d\vartheta_{H^{\#}}}{dt}=\frac{N_{*,\text{tot}}}{N_{\#,\text{tot}}}\left({\dot{\theta }}_{\text{ER}1}-{\dot{\theta }}_{\text{ER}2}\right)\\ \frac{d\vartheta_{\#}}{dt}=\frac{N_{*,\text{tot}}}{N_{\#,\text{tot}}}\left(-{\dot{\theta }}_{\text{ER}1}+{\dot{\theta }}_{\text{ER}2}\right) \end{array}\right.$$ (19)

For lattice oxygen or vacancies, there is Equation 20.

$$\left\{\begin{array}{l} \frac{4}{3}\pi r_{g}^{3}\frac{dC_{O_{O}}}{dt}=-4\pi r_{g}^{2}\Lambda {\dot{\theta }}_{\text{ER}4}\\ \frac{4}{3}\pi r_{g}^{3}\frac{dC_{V_{O}}}{dt}=4\pi r_{g}^{2}\Lambda {\dot{\theta }}_{\text{ER}4} \end{array}\right.$$ (20)

Note that the ion diffusion rate, ${\dot{\theta }}_{\text{ER}4}$ , appears only in Equations 18, 20, which indicates that bulk ion diffusion occurs only on Mn sites, rather than Ca sites. Therefore, Mn is the primary site in this non-catalytic microkinetics, directly affecting the conversion of a grain; Ca is the secondary site accommodating intermediates which assist the reaction process.

The above equations result in a total of eight species evolved by four elementary reactions. The number of independent variables among the species shall be no more than the number of reactions. One restriction is that the sum of all quantities on one type of sites (or the lattices) shall be constant, as Equation 21

$$\left\{\begin{array}{l} \theta_{O^{*}}+\theta_{{\text{OH}}^{*}}+\theta_{{H_{2}O}^{*}}+\theta_{*}=1\\ \vartheta_{H^{\#}}+\vartheta_{\#}=1\\ C_{O_{O}}+C_{V_{O}}=C_{\max}=\rho_{t}\frac{R_{\text{OC}}}{M_{O}} \end{array}\right.$$ (21)

where $\rho_{t}$ is the solid true density, and $M_{O}$ is the molar mass of $O^{2-}$ . Meanwhile, ${\text{OH}}^{*}$ and $H^{\#}$ are always formed or consumed in pairs, giving Equation 22.

$$\vartheta_{H^{\#}}=\frac{N_{*,\text{tot}}}{N_{\#,\text{tot}}}\theta_{{\text{OH}}^{*}}$$ (22)

Consequently, the state of a grain can be determined by four independent variables (for example, $\theta_{O^{*}}$ , $\theta_{{\text{OH}}^{*}}$ , $\theta_{{H_{2}O}^{*}}$ , and $C_{O_{O}}$ ), which equals the number of elementary reactions.

#### 2.3.3 Quasi-steady approximation

Further simplification is obtained from the partial equilibrium assumption, suggesting that the overall reaction rate is controlled by the slowest step, which is Equation 4 in this case; other reversible elementary reactions, Equations 5, 6, 14, are near equilibrium, as concluded by the results in Section 4.2. However, no analytic solution is obtained from the above assumption. Given that the intermediates, ${\text{OH}}^{*}$ and ${H_{2}O}^{*}$ , undergo rapid generation and consumption, resulting in Equation 23

$$\left\{\begin{array}{l} \theta_{{\text{OH}}^{*}}\ll 1\\ \theta_{{H_{2}O}^{*}}\ll 1 \end{array}\right.$$ (23)

an approximate solution is derived as Equation 24

$$\left\{\begin{array}{l} \theta_{*}\approx 1-\frac{C_{O_{O}}}{C_{\max}}\\ \theta_{O^{*}}\approx \frac{C_{O_{O}}}{C_{\max}}\\ \theta_{{H_{2}O}^{*}}\approx \frac{1}{K_{3}}p_{H_{2}O}\left(1-\frac{C_{O_{O}}}{C_{\max}}\right)\\ \theta_{{\text{OH}}^{*}}\approx \sqrt{\frac{1}{K_{2}K_{3}}p_{H_{2}O}\left(1-\frac{C_{O_{O}}}{C_{\max}}\right)} \end{array}\right.$$ (24)

where $K_{i}=k_{+i}/k_{-i}$ is the equilibrium constant.

As only one independent variable ( $C_{O_{O}}$ ) is left among all species, the conversion of a grain is expressed as Equation 25.

$$X_{g}=1-\frac{C_{O_{O}}}{C_{\max}}$$ (25)

Substitute $X_{g}$ into Equations 18, 20, eliminate the rates of Equations 5, 6, 14, and approximate the rate of Equation 4 with its forward rate, giving

$$\frac{dX_{g}}{dt}\approx \frac{1}{1+\frac{\rho_{t}R_{\text{OC}}r_{g}}{3M_{O}\Lambda }}\cdot d_{*}k_{+1}\left(1-X_{g}\right)p_{H_{2}}$$ (26)

The preceding factor of Equation 26 indicates the effect of ion diffusion compared with surface reaction, hereinafter expressed with the symbol Li proposed by Li Z. et al. (2021) and Li (2022), as Equation 27.

$$Li=\frac{1}{1+\frac{\rho_{t}R_{\text{OC}}r_{g}}{3M_{O}\Lambda }}$$ (27)

### 2.4 Particle scale

The rate equation of a grain involves not only the solid conversion, but also the gas partial pressure at the surface, which is not equal for all grains due to their spatial distribution. Grains at the particle surface obtain the most concentrated gas from the atmosphere, while less gas is received by inner grains as it diffuses through the pores, simultaneously consumed by outer grains. Assuming that the grains are evenly distributed throughout the particle’s volume, a radial gas distribution is governed by an equation considering both reaction and diffusion, as Equation 28

$$\left\{\begin{array}{l} \frac{\partial p}{\partial t}=\frac{1}{r^{2}}\frac{\partial }{\partial r}\left(r^{2}D_{\text{eff}}\frac{\partial p}{\partial r}\right)-\omega p\\ {\left.\frac{\partial p}{\partial r}\right|}_{r=0}=0\\ {\left.p\right|}_{r=r_{p}}=p_{s} \end{array}\right.$$ (28)

where $r_{p}$ is the particle radius, and $p_{s}$ is the surface gas partial pressure. The reaction term is derived from the gas–solid stoichiometry, as Equation 29.

$$\omega =\frac{1-\alpha }{\alpha }\frac{\rho_{t}R_{\text{OC}}RT}{M_{O}}\cdot Li\cdot k_{+1}\left(1-X_{p}\right)$$ (29)

The diffusion term is described by the Fick’s Law; the diffusivity shall combine molecular and Knudsen diffusion because the pore radius is smaller than the molecular mean free path length. The particle conversion is the average of all grains, given by Equation 30.

$$\frac{dX_{p}}{dt}=\int_{0}^{r_{p}}Li\cdot k_{+1}\left(1-X_{g}\right)p\cdot 4\pi r^{2}dr/\left(\frac{4}{3}\pi r_{p}^{3}\right)$$ (30)

The governing equation, Equation 28, requires numerical solution due to both p and $X_{g}$ varying along r , which is not applicable to numerous particles in a reactor. Alternatively, a further simplification based on Thiele modulus (Sedghkerdar and Mahinpey, 2015; Yang et al., 2016), proposed in previous studies (Wang et al., 2017; Wang et al., 2021), offers an approximate analytic solution as Equation 31

$$\frac{dX_{p}}{dt}=\eta \cdot Li\cdot k_{+1}\left(1-X_{p}\right)p_{s}$$ (31)

where η is the effectiveness factor of intraparticle gas diffusion, given by Equation 32.

$$\left\{\begin{array}{l} \eta =\frac{3}{\phi^{2}}\left(\phi \coth\phi -1\right)\\ \phi =r_{p}\sqrt{\frac{\omega }{D_{\text{eff}}}} \end{array}\right.$$ (32)

the Thiele modulus ϕ representing the influence of reaction versus diffusion.

### 2.5 Reactor scale

The conversion rate of a particle, as Equation 31, is based on its surface gas partial pressure. Each particle in a fluidized bed moves along a unique trajectory under the fluidization by the inlet gas flow, and is therefore exposed to a gas partial pressure different from other particles. The fluidization behavior is described by the Eulerian–Lagrangian CFD–DEM model in this study, coupling the single-particle reaction kinetics with the particle–fluid motion. The thermal effects are omitted due to the low reduction heat generally observed from various oxygen carriers (García-Labiano et al., 2005; Hallberg et al., 2011), as well as the stable temperature measured during the experiment as Section 3.1.

#### 2.5.1 Particle phase

The particle mass is related to its conversion by Equation 3; consequently, the mass equation of a particle is Equation 33

$$\frac{dm_{p}}{dt}=-m_{p,\text{ox}}R_{\text{OC}}\frac{dX_{p}}{dt}$$ (33)

where $m_{p,\text{ox}}$ is the mass of a fully oxidized particle, and the conversion rate is given by Equation 31.

The translational motion of a particle is evolved by Newton’s Second Law as Equations 34, 35

$$\frac{dx_{p}}{dt}=v_{p}$$ (34)

$$m_{p}\frac{dv_{p}}{dt}=f_{\text{drag}}+f_{\text{coll}}+m_{p}g$$ (35)

and rotational motion as Equation 36

$$I_{p}\frac{dw_{p}}{dt}=T_{\text{coll}}$$ (36)

where $x_{p}$ is the position, $v_{p}$ the velocity, and $w_{p}$ the angular velocity. The drag force, $f_{\text{drag}}$ , is usually expressed in the form of Equation 37

$$f_{\text{drag}}=\frac{\pi }{6}d_{p}^{3}\beta_{p}\left(u_{f}-v_{p}\right)$$ (37)

where $\left(u_{f}-v_{p}\right)$ is the fluid velocity relative to the particle, and $d_{p}$ is the particle diameter; the coefficient $\beta_{p}$ is given by the Koch–Hill model, applicable to both dilute and dense phases (Koch and Hill, 2001). The collision force, $f_{\text{coll}}$ , is decomposed into a normal and a tangential component by the spring-slider-dashpot model (Cundall and Strack, 1979), as Equation 38

$$\left\{\begin{array}{l} f_{N}=\kappa_{N}\Delta x_{N}-\gamma_{N}\Delta v_{N}\\ f_{T}=\min\left\{\kappa_{T}\Delta x_{T}-\gamma_{T}\Delta v_{T},\mu f_{N}\right\} \end{array}\right.$$ (38)

where the subscripts “N” and “T” represent the normal and tangential components, respectively. Both components consist of an elastic force proportional to the overlap Δx , and a damping force depending on the relative velocity Δv , while $f_{T}$ shall not exceed the sliding friction $\mu f_{N}$ . The elastic and damping coefficients, κ and γ , are derived from the restitution coefficient, Young’s modulus, and Poisson’s ratio of the material. Detailed expressions can be found in the computational software (Kloss et al., 2012).

#### 2.5.2 Fluid phase

The gas flow in the reactor is regarded incompressible, described by the governing equations of fluid dynamics. As part of the volume is occupied by the dense particle flow, the governing equations shall involve the fluid volume fraction, denoted as $\alpha_{f}$ . Consequently, the equations of continuity and species transfer are given by Equation 39

$$\left\{\begin{array}{l} \frac{\partial \alpha_{f}}{\partial t}+\nabla \cdot \left(\alpha_{f}u_{f}\right)=-\frac{1}{\rho_{f}V_{\text{cell}}}\sum \frac{dm_{p}}{dt}\\ \frac{\partial \left(\alpha_{f}\rho_{j}\right)}{\partial t}+\nabla \cdot \left(\alpha_{f}u_{f}\rho_{j}\right)-\nabla \cdot \left(\alpha_{f}D_{j}\nabla \rho_{j}\right)=\frac{1}{V_{\text{cell}}}\sum {\dot{m}}_{p,j} \end{array}\right.$$ (39)

where $\rho_{j}$ is the partial density of gas species j . Interphase mass transfer is introduced by the extra source terms, containing the particle reaction rates as Equation 33.

The equation of momentum is expressed as Equation 40

$$\left\{\begin{array}{l} \frac{\partial \left(\alpha_{f}u_{f}\right)}{\partial t}+\nabla \cdot \left(\alpha_{f}u_{f}u_{f}\right)-\nabla \cdot \tau =-\frac{\nabla p}{\rho_{f}}+\alpha_{f}g-\frac{1}{\rho_{f}V_{\text{cell}}}\sum \left(f_{\text{drag}}+\frac{dm_{p}}{dt}u_{f}\right)\\ \tau =\nu_{\text{eff}}\left[\nabla u_{f}+{\left(\nabla u_{f}\right)}^{T}-\frac{2}{3}\left(\nabla \cdot u_{f}\right)I\right] \end{array}\right.$$ (40)

where the interphase drag force given by Equation 37 is considered, along with the momentum carried by the transferred mass. The viscosity $\nu_{\text{eff}}$ contains both laminar and turbulent components, with turbulence solved by the k – ϵ model.

## 3 Experimental and computational setup

### 3.1 Experiment

Validation experiment is conducted on a micro-fluidized-bed thermogravimetric analyzer (MFB–TGA) (Li et al., 2019). A fluidized bed reactor, 3 cm in diameter and 10 cm in height, is placed on an electronic balance, measuring the real-time solid mass change under fluidization throughout the reaction process. More information is provided in the Supplementary Material.

The measurement accuracy is 1 mg, compared with the maximum mass change of 16 mg. Gas inlet and outlet are connected to the reactor with soft tubes to reduce mass fluctuation introduced by the gas circuit. The temperature is maintained constant by an electric furnace and monitored by a K-type thermocouple, which do not contact the reactor. Consequently, the signal from the electronic balance shall accurately present the total solid mass change in the reactor.

The bed inventory consists of silica sand and oxygen carrier particles, whose fluidization properties are listed in Table 2. A bubbling fluidization regime is observed under a gas flux of 1.2 NL/min. Continuous redox cycles are performed by switching the gas among inert (N_2_), oxidizing (O_2_) and reducing (H_2_) components, under different H_2_ concentrations (5 vol%, 10 vol% and 20 vol%) and temperatures (750 °C, 800 °C, 850 °C and 900 °C).

**TABLE 2:** Fluidization properties of particles.

Parameter	Unit	Silica sand	Oxygen carrier
Mass	g	18.0	0.335
Particle number	—	350 000	23 000
Particle diameter	μm	325	215
Particle density	kg/m^3^	2 860	2 800
Young’s modulus	Pa	5 × 10^6^	5 × 10^6^
Poisson’s ratio	—	0.45	0.45
Collision restitution coefficient	—	0.9	0.9
Sliding friction coefficient	—	0.3	0.3

### 3.2 Computation

The simulating conditions in CFD–DEM are identical to those in the MFB–TGA experiment, computational settings and reaction parameters listed in Table 3. Initialization is done by injecting given numbers of sand and fully oxidized oxygen carrier particles, which are immediately packed under gravity. Subsequently, an inert fluidizing gas stream is introduced from the bottom at a given temperature. The gas is switched to a reducing composition after a stable fluidization is achieved, and the reaction simultaneously begins. A mesh-refinement test showing grid-independence is presented in the Supplementary Material. Every case is parallelized into 24 CPU cores and run on a supercomputing center, taking 8 h of real time to progress 1 s of simulation time.

**TABLE 3:** Parameters used in CFD–DEM simulation.

Parameter	Unit	Value
Bed diameter	m	0.03
Bed height	m	0.10
Gas flow rate	NL/min	1.2
Cell number	—	55 125
CFD time step	s	5 × 10^−5^
DEM time step	s	1 × 10^−5^
CMTF8341 true density	kg/m^3^	4 910
CMTF8341 oxygen capacity	—	0.04
CMTF8341 grain radius	nm	75
CMTF8341 specific surface area (BET)	m^2^/g	0.26
Mn site areal density	mol/m^2^	4.60 × 10^−8^
Neighboring site number ( $d_{*}$ )	—	1

The open source software CFDEM®coupling (Kloss et al., 2012) is selected for CFD–DEM computation, which serves as an interface alternately progressing CFD and DEM time steps. CFD is solved by OpenFOAM^®^ with the PISO algorithm; discretization is accomplished through the finite volume method. Particle motion and collision are solved by LIGGGHTS^®^, employing a first-order Euler time integration scheme.

**FIGURE 4:**
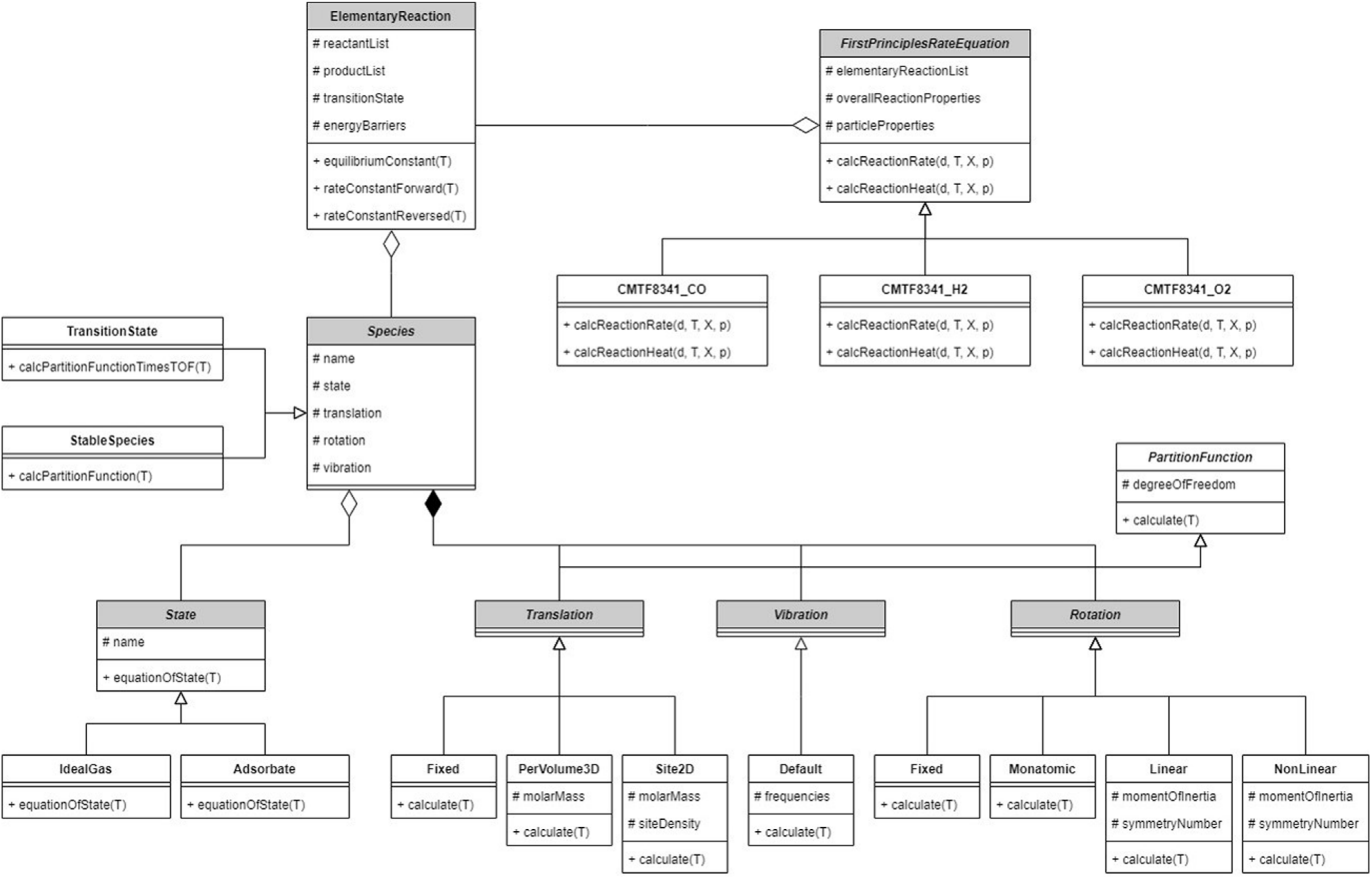
Architecture of the single-particle reaction kinetics program. The abstract class *Species* is used to construct different species and transition states in the reaction path; statistical-mechanical calculation methods are implemented; elementary and overall reactions are constructed from given species.

The reaction kinetics is not implemented in the original software; instead, it is programmed by the authors into an extended package, whose architecture is shown in Figure 4. The overall reaction is described by a class on the top layer, which outputs the particle conversion rate as Equation 31 to CFDEM®coupling; a list of elementary reactions is recorded as its component. The elementary reaction class calculates the rate constants as Section 2.2.2, relying on the reactant and product species along with the transition state. The species class aggregates submodels calculating the equation of state and partition functions; respective model types are selected for every species. DFT calculation is separately performed on the VASP software (Kresse and Furthmüller, 1996) before CFD–DEM computation.

All inputs required by the model, including measured properties, DFT results, reaction equations and submodel types, are provided in a text file during runtime; various overall reactions can be constructed by modifying the inputs, which results in different implementations of the abstract classes.

## 4 Results and discussion

### 4.1 Solid conversion rate

**FIGURE 5:**
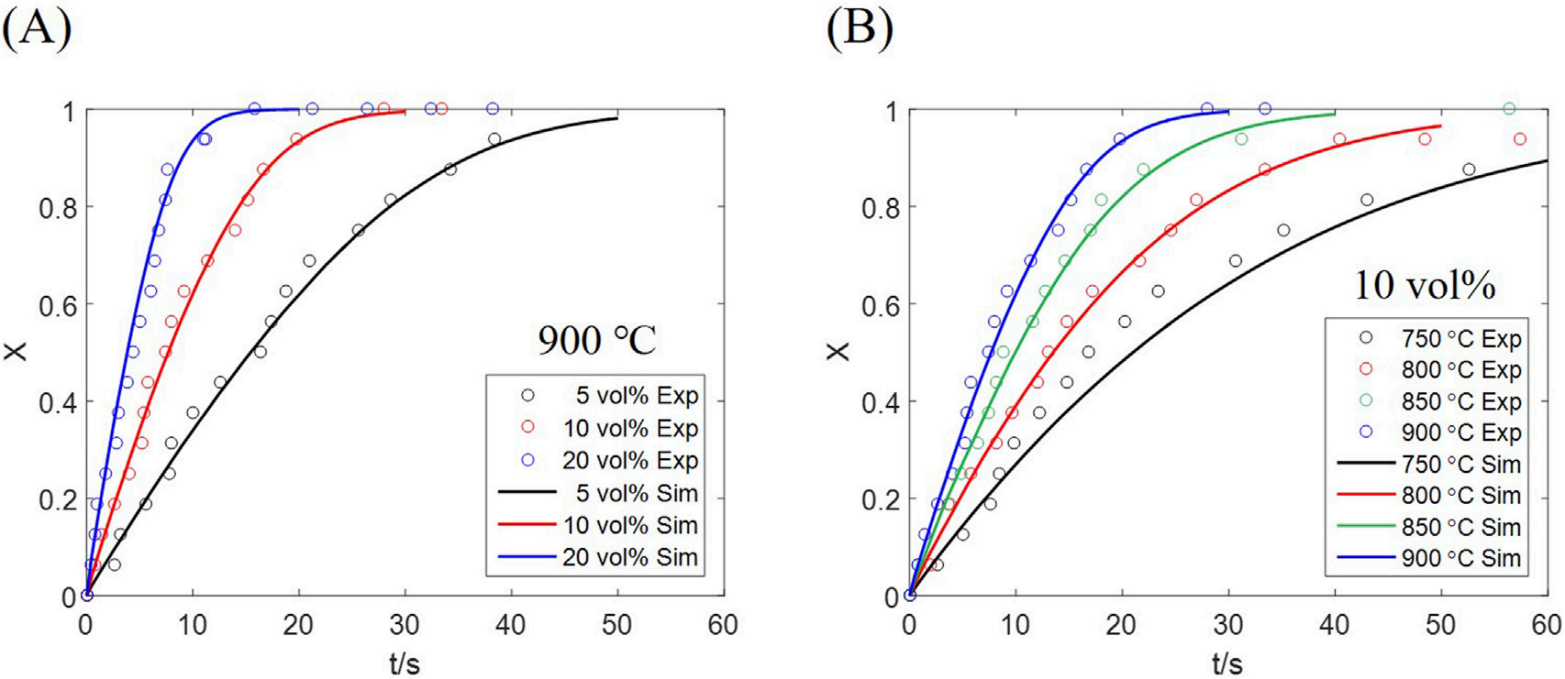
Solid conversion results from computation and experiment at **(A)** different H_2_ concentrations, **(B)** different temperatures.

The conversion of all oxygen carrier particles in the reactor is derived from both computation and experiment as Figure 5. The simulated result represents the average conversion of all particles, corresponding to the experimentally measured conversion of the whole bed inventory. An approximately linear conversion growth is observed in the initial fast stage under every condition, which is transformed to a slow stage as the conversion approaches 0.8. Such phenomenon is affected by the mass transfer resistance of the reactor; as particles near the inlet are converted, more gas is supplied to farther particles, thus compensating the reaction deceleration caused by increased conversion. Therefore, restricting the residence time of particles, to maintain the solid conversion below 0.8, would be beneficial to chemical looping which requires fast redox cycles rather than complete conversion.

The conversion curves under different inlet H_2_ concentrations, at a temperature of 900 °C, are plotted in Figure 5A, which shows that the reaction is accelerated as the inlet concentration increases. An average conversion of 0.8 is reached at ∼8 s for 20 vol% H_2_, while the time is prolonged to ∼15 s for 10 vol% H_2_, and ∼30 s for 5 vol% H_2_. Above results indicate that the fast-stage rate is approximately proportional to the inlet gas concentration, proved by the particle rate equation, Equation 31, where the gas partial pressure appears as an independent factor.

**FIGURE 6:**
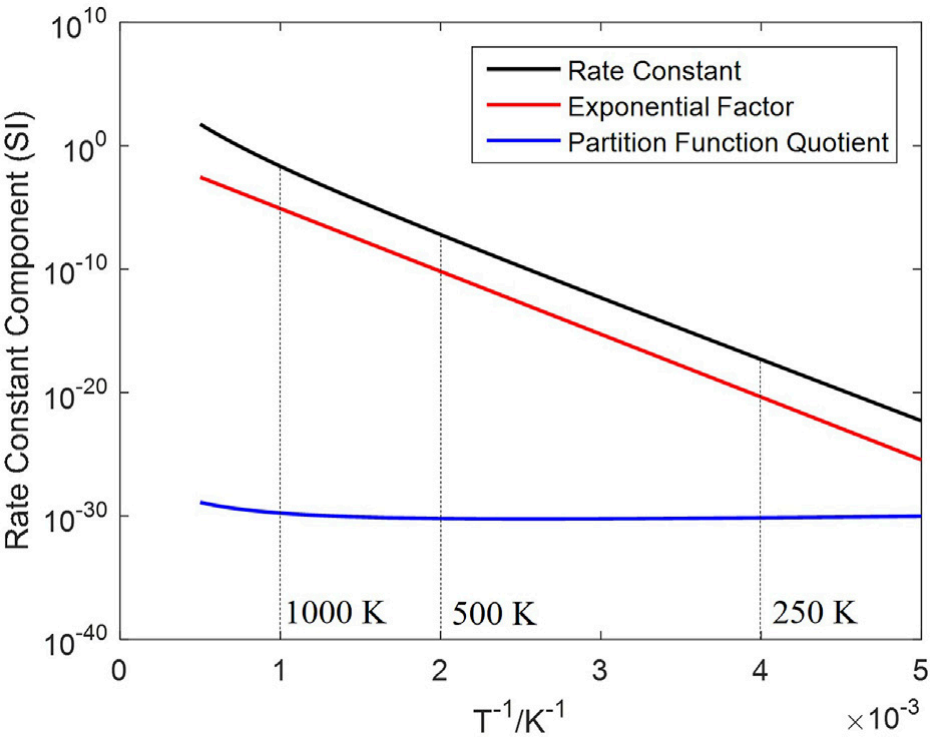
Change of the rate constant and its factors (in logarithm) against inversed temperature.

The effect of temperature under an equal H_2_ concentration (10 vol%) is shown in Figure 5B, a significant rate increase observed at a higher temperature. Temperature has a decisive impact on the rate constant $k_{+1}$ ; two temperature-dependent factors compose the rate constant as Equation 12, including an exponential function of the energy barrier, and a quotient of partition functions. Changes of the rate constant and the two factors against temperature are plotted in Figure 6. The partition function quotient is approximately a power function of the temperature, which grows slower than the exponential factor in the given temperature range; consequently, the rate constant changes in a similar trend to the exponential factor. The impact level of temperature is determined by the energy barrier, $\Delta \varepsilon_{0,+1}$ ; the reduction rate by H_2_ in this study is more sensitive to the temperature compared with CO reduction in a prior study (Wang et al., 2025), which is because a higher energy barrier demands more energy for the reactants to become transition states, thus magnifying the effect of temperature.

**FIGURE 7:**
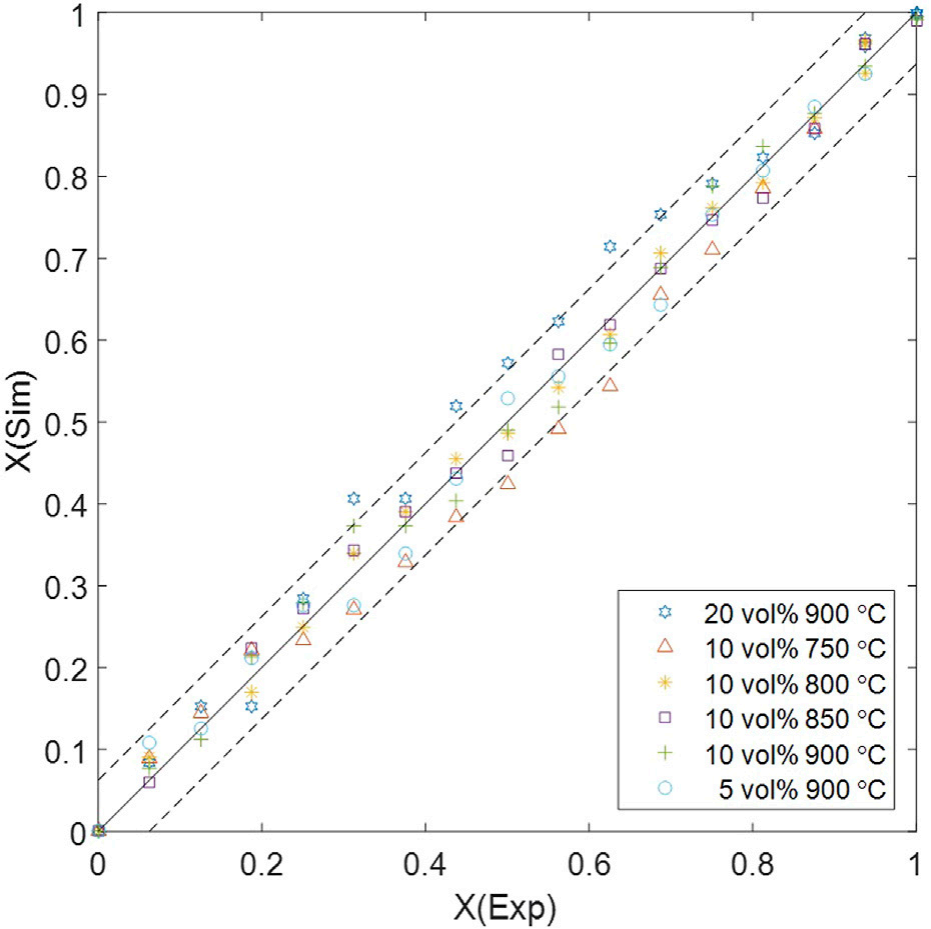
Error margin of computed solid conversion compared with experimental results.

The error margin of solid conversion is determined by the measurement accuracy of MFB–TGA (1 mg), resulting in a solid conversion error of 0.0625. The comparison between computational and experimental results are displayed in Figure 7, showing that the multiscale computation is accurate at most data points. However, exceeded errors are observed under high H_2_ concentration (20 vol%) or low temperature (750 °C) in the middle of the reaction processes. The concentration-related error is mainly introduced by mass transfer, which has a greater impact when a higher inlet concentration leads to a non-uniform distribution; unresolved CFD–DEM omits the fine structure of the particle surface boundary layer, tending to underestimate the mass transfer resistance (Derksen, 2014; Srinivasakannan et al., 2012). Integrating a surface film model into the particle-scale diffusion is a possible way to show the effect of boundary-layer mass transfer; interparticle gas diffusivity in the dense phase should be more accurately modeled considering the sub-scale velocity distribution, which may need validation by particle-resolved DNS–DEM before application to CFD–DEM (Wang et al., 2023). The temperature-related error depends on the energy barrier $\Delta \varepsilon_{0,+1}$ , which is generated during DFT calculation; an increased energy barrier results in a more temperature-sensitive reaction rate. The energy error mainly depends on the functional selection; as different functionals are suitable for different systems, accuracy can be improved by choosing an appropriate functional for calculation (Kim et al., 2013). Besides, various energy correction schemes have been developed against systematic errors, based on error analysis versus experimental data, or functional dependence on energies (Christensen et al., 2015).

### 4.2 Microkinetics with bulk diffusion

The areal density of Mn sites ( Λ ) and the number of neighboring sites ( $d_{*}$ ) are hypothetic parameters based on Langmuir’s adsorption model, which are not yet measurable; therefore, they are regarded as adjustable parameters to be assigned from the experimental results. The Mn site areal density (4.60 × 10^−8^ mol/m^2^) has been determined in a previous study (Wang et al., 2025), where the single-site mechanism of CMTF8341 reduced by CO, occurring on the same Mn site, was modeled; Λ appeared as the only unknown parameter, and was adjusted according to the CO-reduction results. The number of neighbors is valued as 1 so that the computational results fit those from the experiment. This result indicates that every Mn site is paired to exactly one nearest Ca site, and every dual-site reaction occurs on a predetermined pair of sites.

**FIGURE 8:**
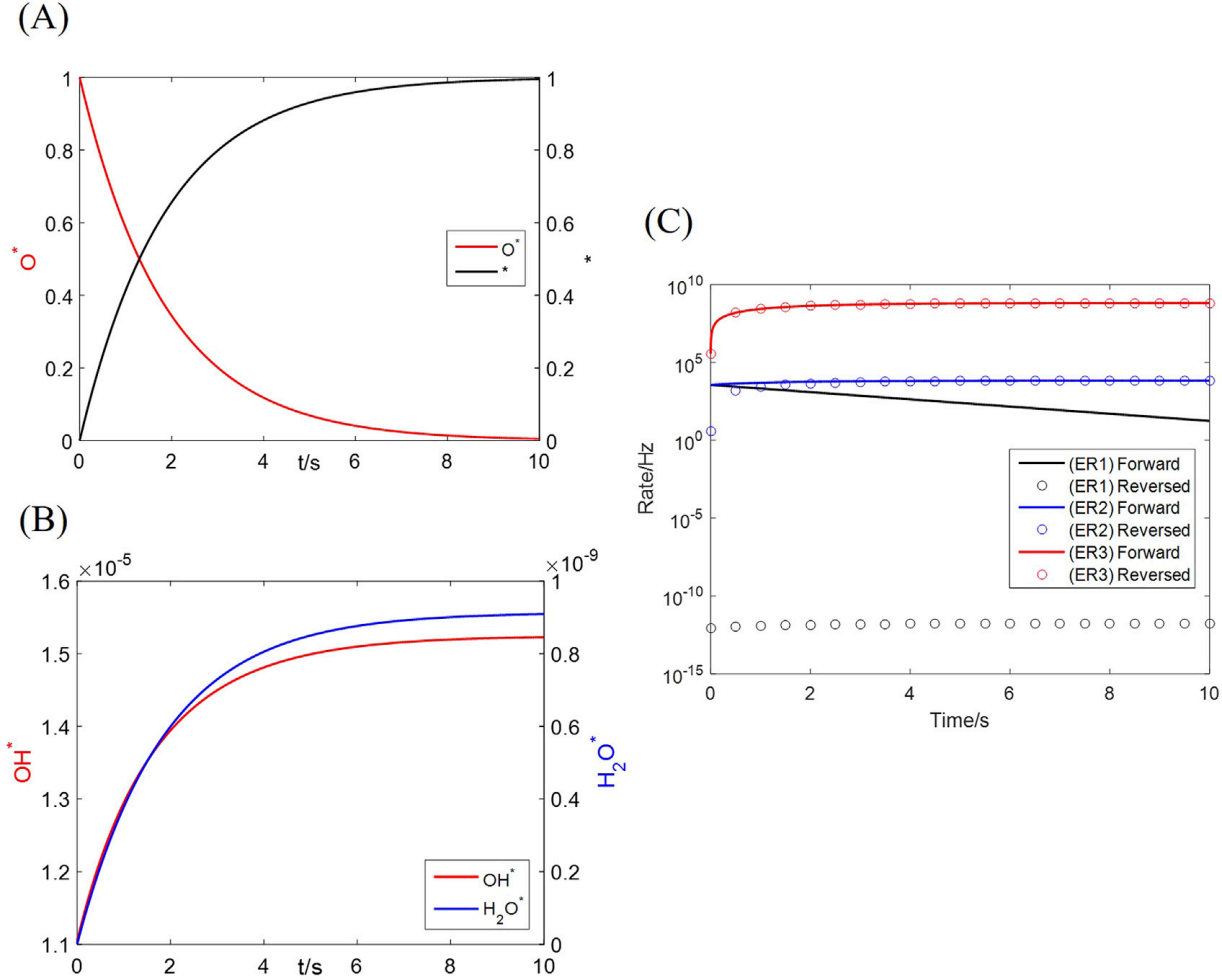
Numerical solution to the dual-site microkinetics with bulk diffusion. **(A)** Coverages of reactant and product species; **(B)** Coverages of intermediates; **(C)** Forward and reversed elementary reaction rates.

The microkinetics as Equations 18–20 is originally solved as an ordinary differential equation set; an example condition of 20 vol% H_2_, 1 vol% H_2_O and 900 °C is adopted in this section. The coverages of all species on the primary (Mn) site vary as Figures 8A,B. The major components are the reactant ( $O^{*}$ ) and product ( * ), which undergo substantial conversion throughout the reaction process; coverages of the minor intermediates ( ${\text{OH}}^{*}$ and ${H_{2}O}^{*}$ ) grow in the same trend, but are restricted in an order of 10^–9^ and 10^–5^, respectively. The forward and reversed rates of every elementary reaction change as Figure 8C. Every elementary reaction starts with a positive net rate, converting $O^{*}$ to ${\text{OH}}^{*}$ , ${H_{2}O}^{*}$ , and * in order. An equilibrium is established for reaction Equation 6 immediately after the reaction begins, owing to a high turnover frequency of the H_2_O adsorption–desorption process. As the reaction further progresses, Equation 5 also approaches equilibrium at ∼1 s as its reversed rate increases. Consequently, Equations 5, 6 can be accurately described by the partial equilibrium assumption. Above results have proved that Equation 4 is the rate-determining step, which is far from equilibrium throughout the reaction. The approximated coverages of surface species in Equation 24 are thus verified, leading to an analytic grain conversion rate as Equation 26.

The overall reaction Equation 17 has an equilibrium point when elementary reactions Equations 4, 5, 6, 14 are all equilibrated, as required by the principle of detailed balance. Let Equations 4, 5, 6, have zero net rates in Equations 5, 8 and 9, combined with the ion-diffusion equilibrium as Equation 15, resulting in Equation 41.

$$\frac{p_{H_{2}O}C_{V_{O}}}{p_{H_{2}}C_{O_{O}}}=\frac{\frac{q_{H_{2}O}^{0}}{V}q_{*}^{0}}{\frac{q_{H_{2}}^{0}}{V}q_{O^{*}}^{0}}\exp\left(-\frac{\Delta \varepsilon_{0,\text{overall}}}{k_{B}T}\right)$$ (41)

**FIGURE 9:**
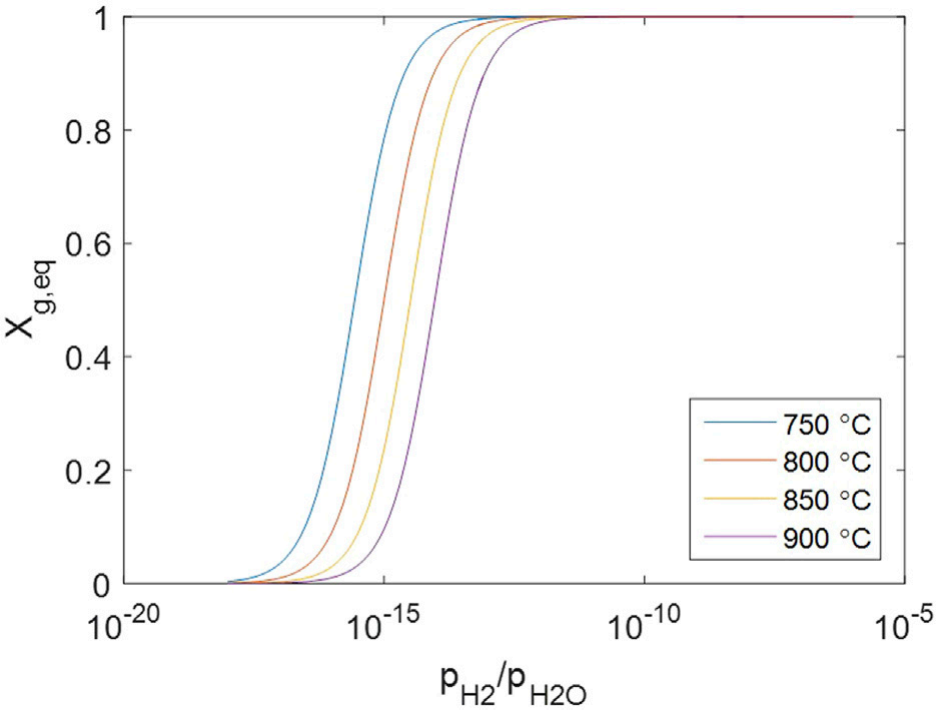
Change of equilibrium solid conversion against gas concentrations.

Note that the solid concentrations are involved because crystal defects are treated as non-localized systems, which is not the case for perfect crystal species in other common reactions. The equilibrium solid conversion changes against gas concentrations as Figure 9, suggesting that the solid is almost fully converted under normal conditions when both $p_{H_{2}}$ and $p_{H_{2}O}$ have an order of kPa–MPa. Only under extreme conditions when $p_{H_{2}}/p_{H_{2}O}<10^{-13}$ , typically when the gas reactant is depleted due to insufficiency, can the solid achieve incomplete conversion.

**FIGURE 10:**
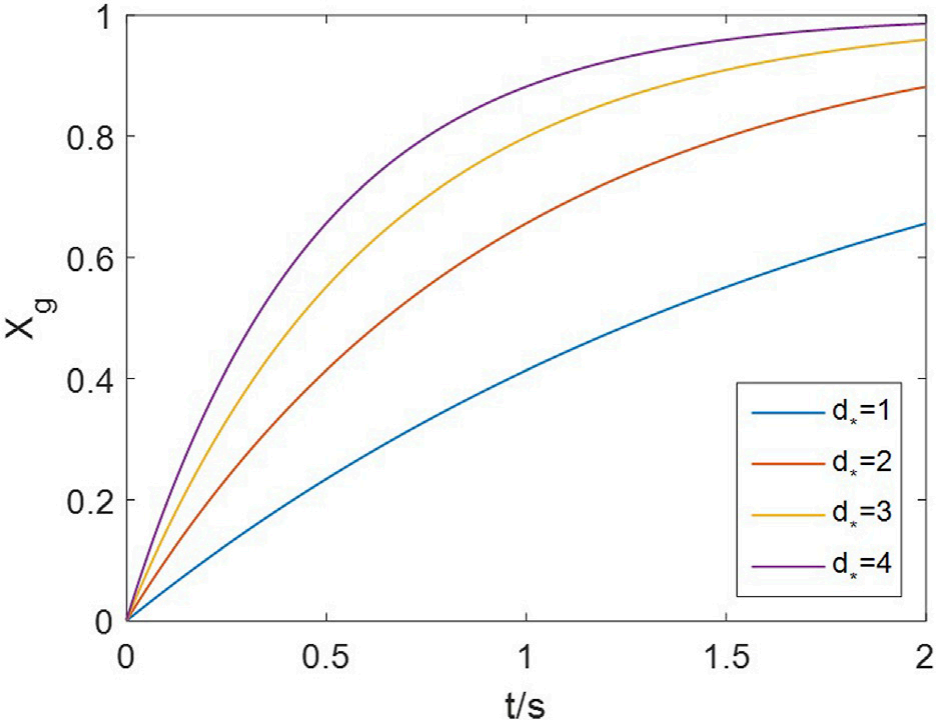
Effect of neighboring-site number on the grain’s conversion rate (20 vol% H_2_, 900 °C).

The number of neighboring sites ( $d_{*}$ ), in every dual-site elementary reaction rate, is a positive integer depending on the surface site distribution. With (4) being the rate-determining step, $d_{*}$ appears as an independent factor in the grain’s rate as Equation 26. Figure 10 shows the grain’s conversion curve at 20 vol% H_2_ and 900 °C, as $d_{*}$ increases from 1 to 4. Rates proportional to $d_{*}$ are observed in the early stage when $X_{g}<0.5$ . Consequently, the proportional effect of $d_{*}$ propagates to the particle and reactor scale, given that all grains and particles share the same $d_{*}$ . A proper value of $d_{*}$ should fit every concentration and temperature, as it remains constant under all operating conditions.

### 4.3 Particle diversity

**FIGURE 11:**
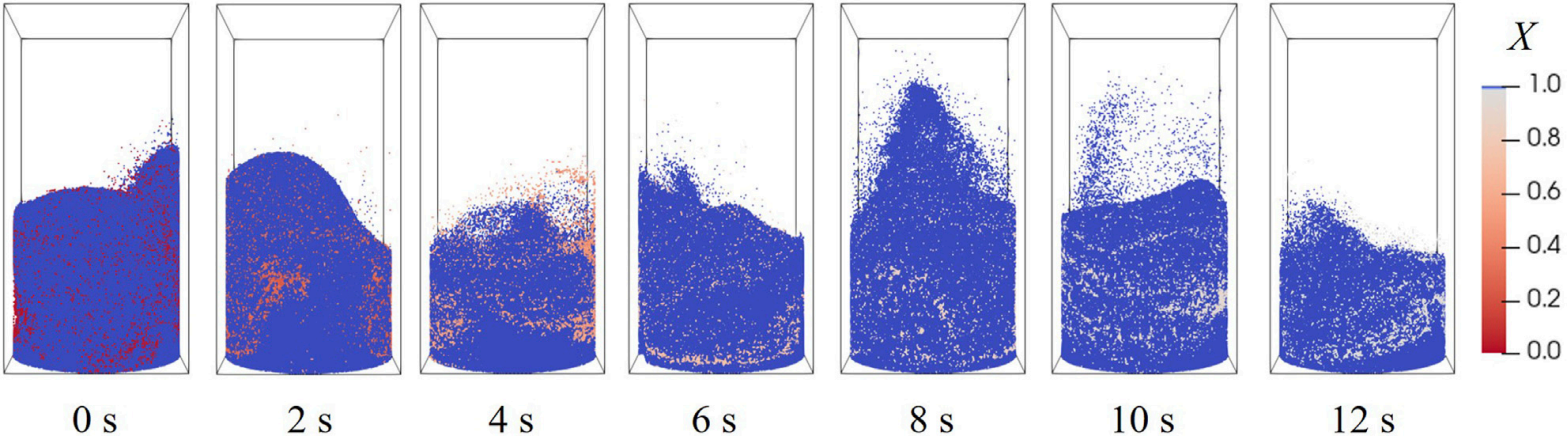
Particle location and conversion distributions at different moments.

The evolution of particle distribution at 900 °C, 20 vol% H_2_, is illustrated in Figure 11. The moment of 0 s is the starting point of reaction, when a stable bubbling fluidization has been established. Particles in the center are elevated by the gas stream, and subsequently fall along the walls under gravity; the bed height fluctuates around 3 cm as the void fraction of the bed inventory zone changes. The conversions of oxygen carrier particles are marked by color in Figure 11, where only a minor difference exist among all particles, suggesting that an even mixture is accomplished by fluidization.

**FIGURE 12:**
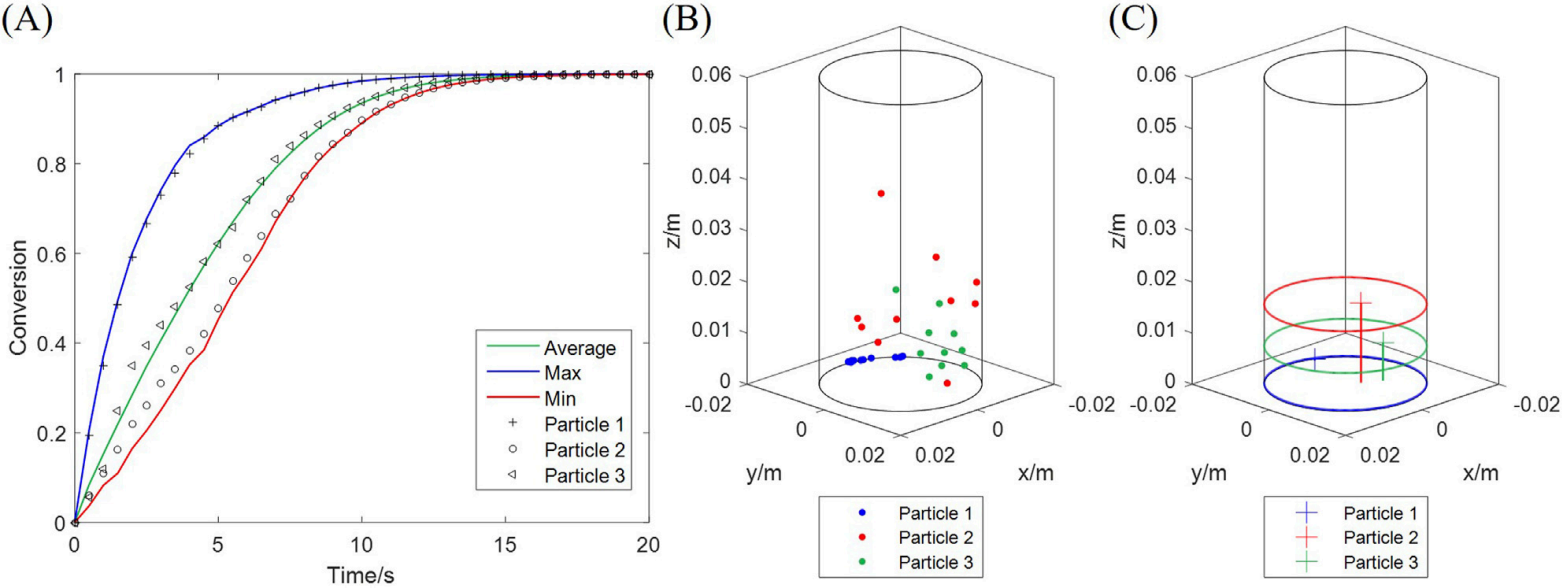
Conversion of three typical particles undergoing fast (particle 1), slow (particle 2) and average (particle 3) reactions. **(A)** Conversion curves; **(B)** Positions at different moments; **(C)** Average positions during the initial 5 s.

Both the computational and experimental results in Section 4.1 display the overall solid conversion, which equals the average of all particles. However, the conversion of a single particle may deviate from the average. The average, maximum and minimum conversions among all particles are plotted in Figure 12A, along with three typical particles, at 20 vol% H_2_, 900 °C. Particle 3 undergoes an average-rate reaction, while Particle 1 is faster and Particle 2 slower. These particles are exposed to particular H_2_ concentrations at every moment, determined by their unique trajectories during fluidization. The positions of the three particles, sampled during the initial fast stage of the reaction (0–5 s), are presented in Figures 12B,C. Particle 1 has the lowest average position in height, and Particle 2 the highest, which indicates that Particle 1 is exposed to a higher concentration of H_2_ than Particles 3 and 2.

**FIGURE 13:**
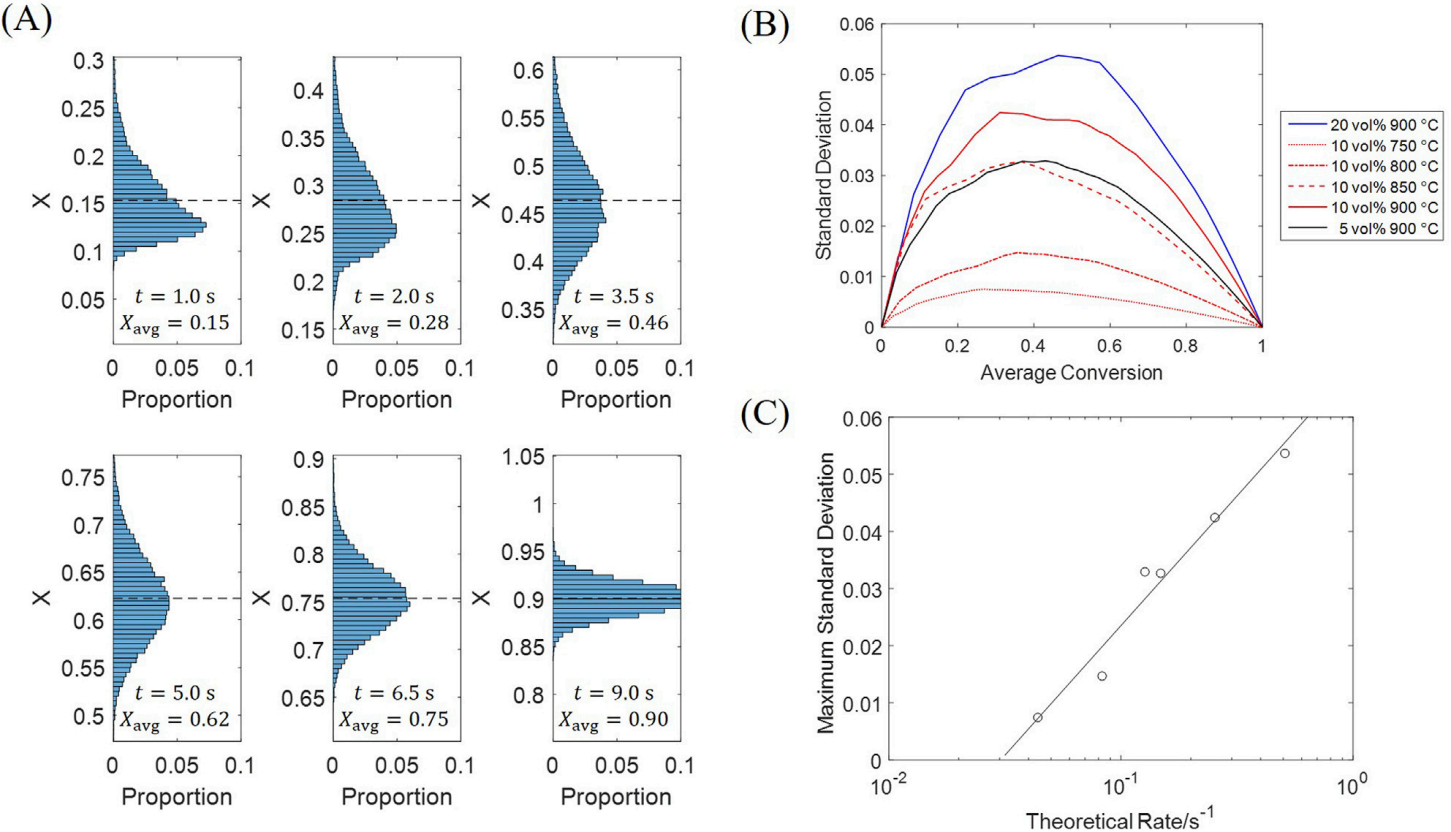
Standard deviation of particle conversion at different moments. **(A)** Histograms; **(B)** Standard deviation against average conversion; **(C)** Maximum standard deviation against theoretical reaction rate.

A deviation among particle conversions is accumulated during the initial fast stage, with the fastest rate being 2–3 times the slowest rate, which subsequently decreases as the particles are converted. Histograms in Figure 13A display the particle conversion distributions at different average conversions, which all show a normal distribution scheme. Besides, the particle conversion distribution is biased to the lower side in the beginning, which is because most H_2_ is consumed by the few particles near the inlet, while other particles only obtain a low H_2_ concentration.

The standard deviation reaches its maximum at an average conversion of 0.3–0.5, under all tested conditions as Figure 13B; a greater standard deviation is observed for a condition with a faster reaction rate (given by Figure 5). The maximum standard deviation can be selected as a quantitative indicator of particle diversity, which is plotted against the theoretical reaction rate in Figure 13C. The theoretical reaction rate is calculated from Equation 31, when the particle has a conversion of zero and obtains an inlet gas concentration, acting as a superior limit which leads to a rate between 0% and 100% of this limit for every single particle. Results show that the maximum standard deviation is approximately linear against the logarithm of theoretical rate. As the fluidization process provides the spatial H_2_ concentration distribution with a similar pattern under all conditions, consequently, a greater deviation is formed at a faster theoretical rate. Such diversity of particles decelerates the overall reaction, because more gas is involved in a slower reaction stage with the highly-converted particles; the utilization efficiency of the solid material is thus affected, which shall be compensated in the system design.

### 4.4 Intraparticle gas diffusion

**FIGURE 14:**
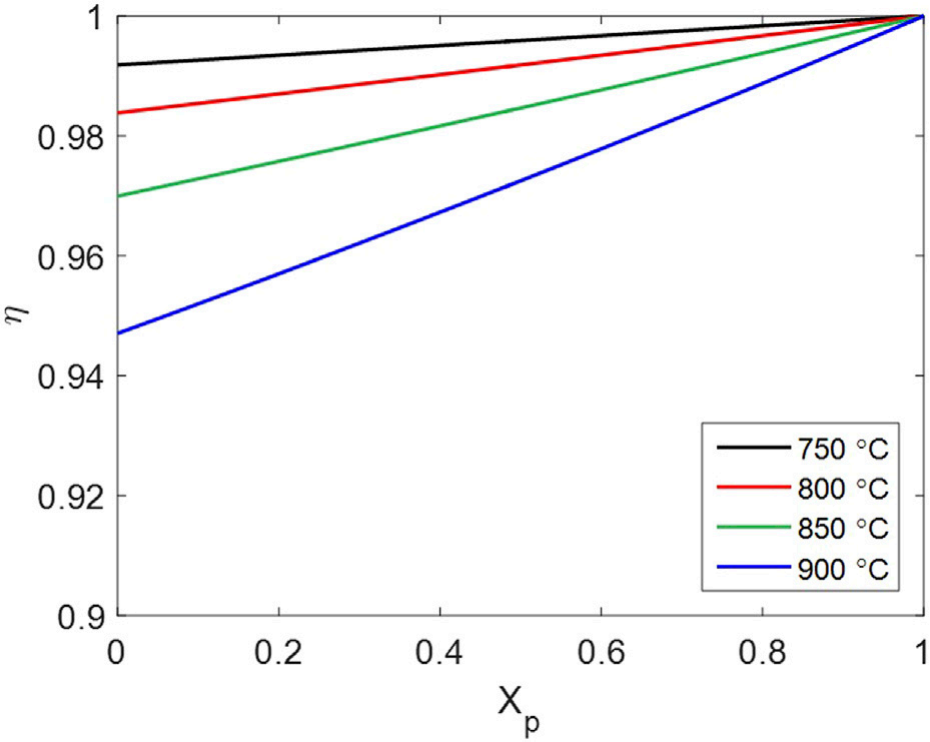
Effectiveness factor of intraparticle gas diffusion changing with particle conversion.

The influence of intraparticle gas diffusion is represented by the effectiveness factor η , defined as Equation 32, which changes against the particle conversion as Figure 14. The effectiveness factor at every temperature remains above 0.9 throughout the reaction process, indicating that the conversion rate of the overall particle is approximately equal to that of a grain at the particle surface in the studied cases. A larger Thiele modulus results in a smaller effectiveness factor; thus, η increases with solid conversion, given that the rate decreases during the reaction; meanwhile, η decreases with temperature, owing to the rate constant ( $k_{+1}$ ) positively correlated to the temperature. Generally, the reduction of CMTF8341 by H_2_ has a relatively low rate compared with gas diffusion, so intraparticle gas diffusion has a minor impact among all processes in the multiscale framework.

### 4.5 Overall reaction order

The overall rate equation derived from quasi-steady approximation, Equation 26, is first-order for the solid species, as the solid conversion appears as a factor of $\left(1-X_{g}\right)$ . This feature is mainly determined by the rate-determining step, Equation 4, where only one solid reactant ion ( $O^{*}$ ) is involved. Contrarily, previous studies on H_2_ reduction kinetics (Li and Li, 2024; Li, 2022; Li Z. et al., 2021) have generally adopted the symmetrical dissociation mechanism as Equations 42–44

$$H_{2}\left(g\right)+2O^{*}⇌2{\text{OH}}^{*}$$ (42)

$$2{\text{OH}}^{*}⇌{H_{2}O}^{*}+O^{*}$$ (43)

$${H_{2}O}^{*}⇌H_{2}O\left(g\right)+*$$ (44)

where the H_2_ molecule is dissociated on two identical sites ( * ) and combined with two oxygen atoms. Repeating the surface- and grain-scale derivations, with the rate-determining step being Equation 42, results in a second-order overall rate equation as Equation 45

$$\frac{dX_{g}}{dt}=Li\cdot d_{*}k_{+1}{\left(1-X_{g}\right)}^{2}p_{H_{2}}$$ (45)

which significantly varies from the first-order rate under dual-site dissociation Equation 4.

Experimental validation in Section 4.1 has agreed with the first-order rate equation, along with the dual-site dissociation mechanism. Dual-site microkinetics lead to asymmetrical impacts on bulk diffusion, which only occurs on primary sites (Mn) rather than secondary sites (Ca). Therefore, the atom-scale reaction mechanism may have crucial impacts on the mesoscale mass transfer and macroscopic kinetics.

### 4.6 Computational cost for scale-up systems

**FIGURE 15:**
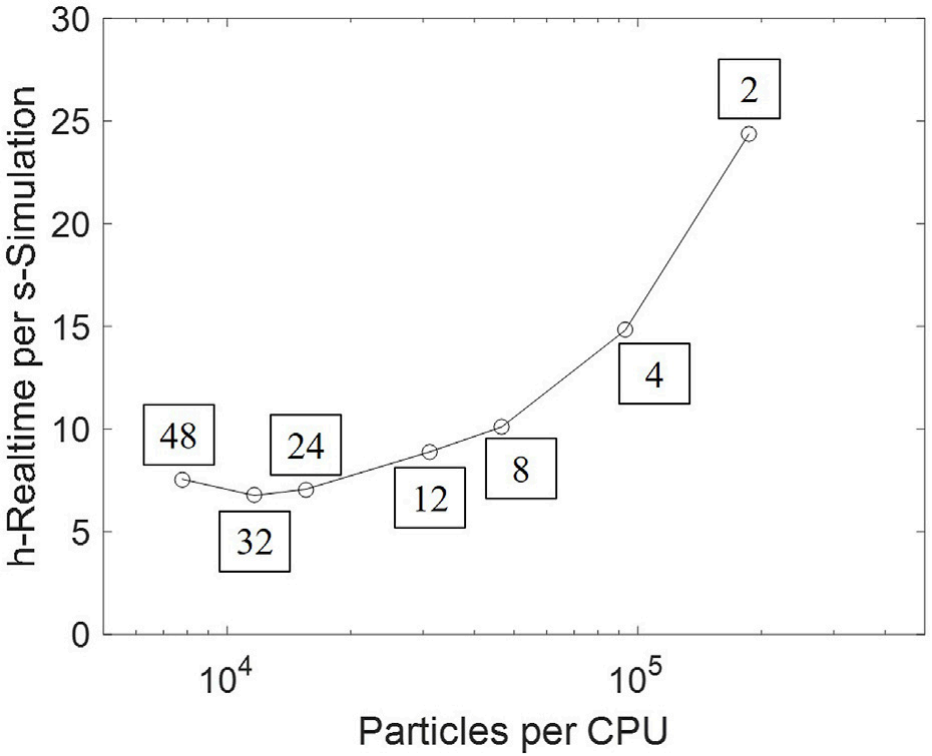
Computational time varying against the number of particles per processor. The processor number is tagged beside each data point.

The heavy computational cost has been an obstacle to scaling-up CFD–DEM simulation to industrial-scale reactors. Parallel computing accelerates computation by independently handling local interactions. A scale-up test is conducted based on the setup as Section 3.2, by varying the number of processors between 2 and 48, thus changing the number of particles per processor. The real time cost per second of simulation time is plotted in Figure 15. An approximately proportional acceleration is obtained in lowly-parallelized cases. However, extra cost is introduced by interprocess communication; the computational speed thus remains stable and subsequently slows down as more processors are employed.

Therefore, further acceleration approaches shall provide essential improvements, for either computation or the DEM model itself. Graphical processing units (GPU), which are especially applicable to batches of simple computation, can be used to handle the massive particle collision (Lu, 2022). Existing investigation on modeling improvements include: (1) the coarse-grain model, which significantly reduces particles in the system, by combining multiple particles into a parcel (Sakai et al., 2014); (2) machine learning algorithms, which replaces direct force calculation with a prediction–correction updating (Lu et al., 2021). These modeling treatments have partially sacrificed the accuracy of fluidization; however, given the minor effect of particle diversity (as Section 4.3), such simplification would be acceptable, provided that the fluidization regime is maintained.

## 5 Conclusion

A multiscale model is developed regarding the whole process of non-catalytic heterogeneous reactions in a fluidized bed. Physical and chemical processes spanning across various scales are involved, including the atom-scale elementary reaction path, the surface-scale dual-site microkinetics, the grain-scale asymmetrical bulk diffusion, the particle-scale gas diffusion, and the reactor-scale fluidization behavior. The model is applied to the reduction of CMTF8341 by H_2_ and validated by the MFB–TGA experiment, revealing the effects of inlet gas concentration and temperature on the macroscopic reaction kinetics.

The prediction of overall reaction rate strongly relies on the reaction path from DFT calculation, whose results suggest a dual-site dissociation for H_2_ molecules, resulting in a first-order overall rate equation compared with the second-order symmetrical dissociation mechanism. A mean-field microkinetics regarding active site pairs is established, every site connected to an equal number of neighbors on the periodical surface, forming the same number of equivalent reaction paths. Furthermore, the dual-site microkinetics is coupled with bulk diffusion in an asymmetrical approach, where the primary site exchanges ions with inner lattices, while the secondary site only accommodates intermediates to assist surface reactions. The neighboring-site number is currently a hypothetic parameter; scanning transmission electron microscopic (STEM) and scanning tunneling microscopic (STM) observation are potential ways to justify corresponding assumptions.

The rigorous theoretical model is based on physical parameters instead of experimental fitting, thus reducing the experimental costs when applied to other reaction systems. An extensible software package is developed for mesoscopic models and integrated into open source CFD–DEM software; generalization to other heterogeneous reactions can be accomplished by inputting DFT results along with elementary reaction equations and instrumentally measured properties. This work is conducted on a lab-scale reactor, while the computational cost is an obstacle to scaling the model to industrial-scale systems; potential methods including coarse-graining models and GPU acceleration may be involved in relevant future studies.

## Data Availability

The raw data supporting the conclusions of this article will be made available by the authors, without undue reservation.

## Glossary

$C_{i}$: Concentration of lattice species *i*, mol/m^3^
$C_{\max}$: Maximum concentration of lattice oxygen, mol/m^3^
$d_{*}$: Number of neighboring sites of a Mn site
$d_{\#}$: Number of neighboring sites of a Ca site
$d_{p}$: Particle diameter, m
$D_{\text{eff}}$: Effective intraparticle diffusivity, m^2^/s
$D_{j}$: Diffusivity of gas species *j*, m^2^/s
$f_{\text{drag}}$: Drag force, N
$f_{N}$: Normal collision force, N
$f_{\text{coll}}$: Collision force, N
$f_{T}$: Tangential collision force, N
g: Gravitational acceleration, m/s^2^
h: Planck constant, J·s
J: Flux of oxygen on the grain surface, mol/(m^2^·s)
$k_{B}$: Boltzmann constant, J/K
$k_{+}$: Rate constant of an elementary reaction (forward)
$k_{-}$: Rate constant of an elementary reaction (reversed)
Li: Li number
m: Mass of oxygen carrier in the reactor, kg
$m_{\text{ox}}$: Mass of fully-oxidized oxygen carrier, kg
$m_{p}$: Mass of a particle, kg
$m_{p,\text{ox}}$: Mass of a fully-oxidized particle, kg
$m_{\text{re}}$: Mass of fully-reduced oxygen carrier, kg
${\dot{m}}_{p,j}$: Mass transfer rate of gas species *j*, kg/s
$M_{O}$: Molar mass of oxygen atoms, kg/mol
$N_{*,\text{tot}}$: Number of Mn sites on a surface
$N_{\#,\text{tot}}$: Number of Mn sites on a surface
$N_{*\#,\text{tot}}$: Number of Mn–Ca site pairs on a surface
$N_{i}$: Number of surface species *i*
$\dot{N}$: Net reaction frequency on the surface, s^−1^
p: Gas partial pressure field, Pa
$p_{i}$: Partial pressure of gas species *i*, Pa
$p_{s}$: Gas partial pressure at particle surface, Pa
$q_{i}^{0}$: Partition function of species *i* based on the reference energy
$q_{\text{TS}}^{0,‡}$: Partition function of transition state excluding dissociation
r: Radius coordinate, m
$r_{g}$: Grain radius, m
$r_{p}$: Particle radius, m
R: Ideal gas constant, J/(mol·K)
$R_{\text{OC}}$: Oxygen capacity of oxygen carrier
t: Time, s
T: Reaction temperature, K
$u_{f}$: Fluid velocity, m/s
$v_{p}$: Particle velocity, m/s
$\Delta v_{N}$: Normal relative particle velocity, m/s
$\Delta v_{T}$: Tangential relative particle velocity, m/s
V: Gas volume, m^3^
$V_{\text{cell}}$: Cell volume, m^3^
$x_{p}$: Particle position, m
$\Delta x_{N}$: Normal particle overlap, m
$\Delta x_{T}$: Tangential particle overlap, m
X: Conversion of oxygen carrier in the reactor
$X_{g}$: Conversion of a grain
$X_{p}$: Conversion of a particle
α: Particle porosity
$\alpha_{f}$: Volume fraction of fluid phase
$\beta_{p}$: Drag force coefficient, kg/(m^3^·s)
$\gamma_{N}$: Normal damping coefficient, N·(m/s)^−1^
$\gamma_{T}$: Tangential damping coefficient, N·(m/s)^−1^
δ: Atomic number in CaMn_0.375_Ti_0.5_Fe_0.125_O_3−*δ*_
$\Delta \varepsilon_{0,+}$: Energy barrier of an elementary reaction (forward), J
$\Delta \varepsilon_{0,-}$: Energy barrier of an elementary reaction (reversed), J
η: Effectiveness factor of intraparticle gas diffusion
$\theta_{i}$: Coverage of Mn-site surface species *i*
$\dot{\theta }$: Net reaction rate per Mn site, s^−1^
$\vartheta_{i}$: Coverage of Ca-site surface species *i*
$\kappa_{N}$: Normal spring coefficient, N/m
$\kappa_{T}$: Tangential spring coefficient, N/m
Λ: Areal density of Mn sites, mol/m^2^
$\nu_{\text{eff}}$: Effective kinematic viscosity, m^2^/s
$\rho_{f}$: Fluid density, kg/m^3^
$\rho_{j}$: Density of gas species *j*, kg/m^3^
$\rho_{t}$: Particle true density, kg/m^3^
τ: Viscous stress tensor, m^2^/s^2^
ϕ: Thiele modulus
ω: Intrinsic rate constant for gas, s^−1^
