# Supplementary material for: Multiscale reaction model coupling dual-site microkinetics with bulk diffusion and CFD–DEM for a perovskite oxygen carrier
Source: Front Chem. 2025 Sep 4;13:1656180. doi: 10.3389/fchem.2025.1656180 (PMC12445058; doi:10.3389/fchem.2025.1656180)
Supplement: Supplementary file 1 [file DataSheet1.docx]

Supplementary Material to

Multiscale reaction model coupling dual-site microkinetics with bulk diffusion and CFD–DEM for a perovskite oxygen carrier

Ruiwen Wang^1^, Zhenshan Li^1*^, Lei Liu^2^

^1^Key Laboratory for Thermal Science and Power Engineering of Ministry of Education, Department of Energy and Power Engineering, Tsinghua University, Beijing, China

^2^Hunan Engineering Research Center of Clean and Low-Carbon Energy Technology, School of Energy Science and Engineering, Central South University, Changsha, China

*** Correspondence:**Zhenshan Li
lizs@tsinghua.edu.cn

# MFB–TGA Experiment

## Experimental System


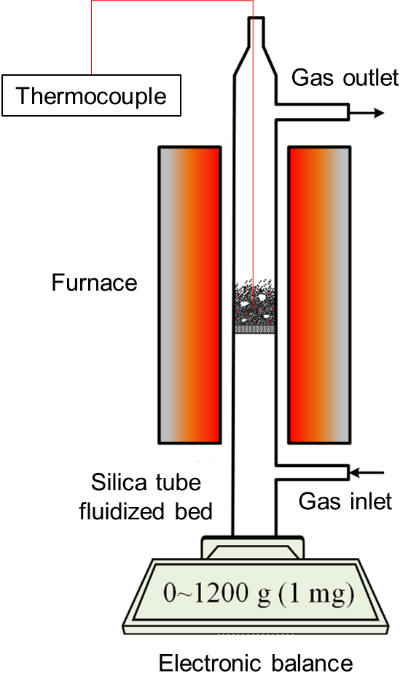


**Supplementary Figure 1.** Schematic diagram of the MFB–TGA experimental system.

A schematic diagram of the MFB–TGA experimental system is shown in **Supplementary Figure 1**. The fluidized bed reactor is supported by a silica tube; the bed inventory is located on a gas distributor in the middle. Different inlet gases are provided by gas cylinders, which are switched via an electromagnetic valve, controlled by a desktop computer.

A blank test with no reactions is first conducted, to ensure the measuring stability. Silica sand (with no oxygen carrier particles) is used as the solid material, and pure N_2_ as the fluidizing gas. The mass fluctuation shall be within ±1 mg under stable fluidization when heated to predetermined temperatures. Afterward, the inlet gas is switched between two different components, during which the mass signal shall also be stable.

## Test Results over Multiple Redox Cycles

Tests over redox cycles are conducted by switching the gas in a looping order of: blank–reduction–blank–oxidation, where the blank phases use pure N_2_ to eliminate the previous gas. Different volume fractions of H_2_ (5%, 10%, 20%) and O_2_ (5%, 10.5%, 15%, 21%) are adopted in different cycles. Tests under every condition is repeated (at least 3 times) and averaged for model validation.

The mass signal over several redox cycles is as **Supplementary Figure 2**. The mass is observed to rise and fall in a stable range (−4% to 0%) over multiple cycles, which shows that the oxygen carrier is recovered through oxidation, before another reduction process. Averaged results under every condition can be found in our prior experimental study **(Liu L. et al., 2021)**.


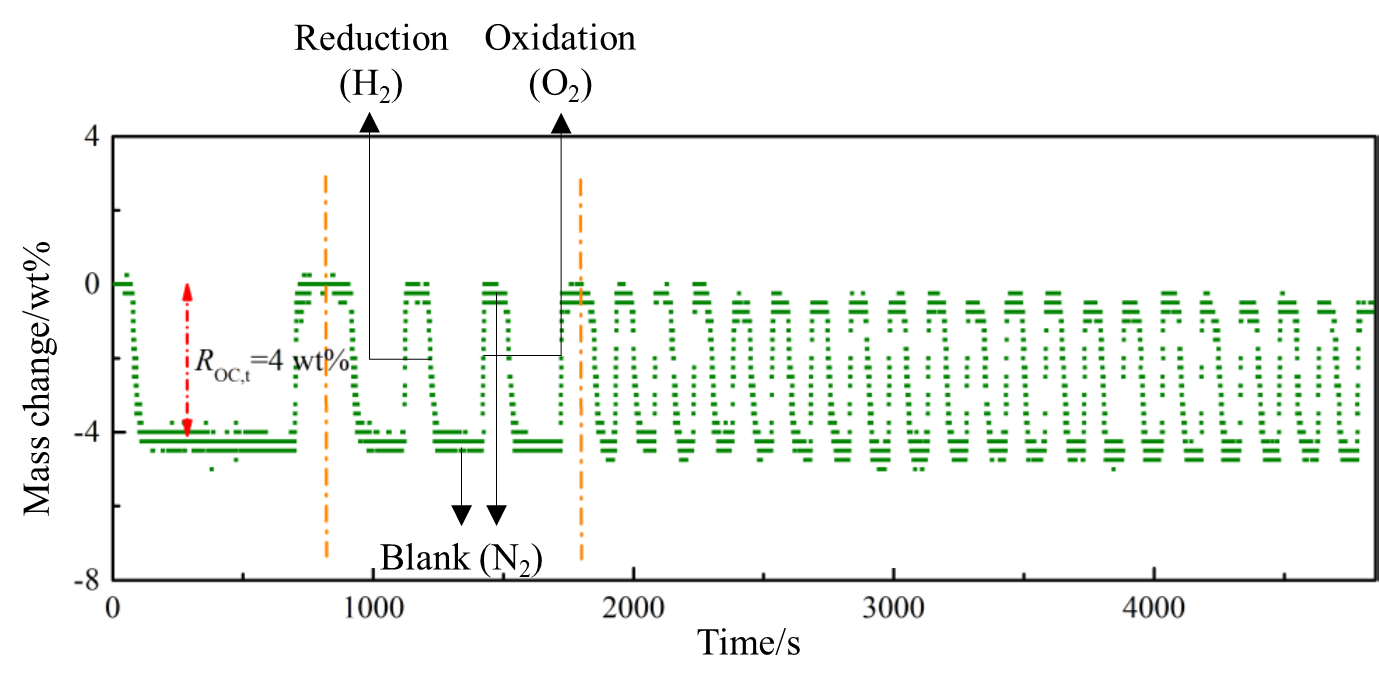


**Supplementary Figure 2.** Mass signal over continuous redox cycles.

# CFD–DEM Meshing

The mesh used in CFD–DEM is illustrated in **Supplementary Figure 3(A)**, which is uniformly divided along the height; the cross section is divided into five blocks including a square in the center, so that the hexahedral cells do not have too small angles. The edge lengths of the cells are set to 0.94–1.59 mm, equivalent to 2.9–4.9 times the mean particle diameter (325 μm), which meets the requirement of unresolved CFD–DEM (3–5 times).

A denser mesh as **Supplementary Figure 3(B)** is also constructed for a refinement test, with a cell number of 90720 (increased by 65% than the original mesh), and edge lengths of 0.79–1.32 mm (2.4–4.1 times the particle diameter). The testing condition is selected as 20 vol% H_2_, 900 ℃.


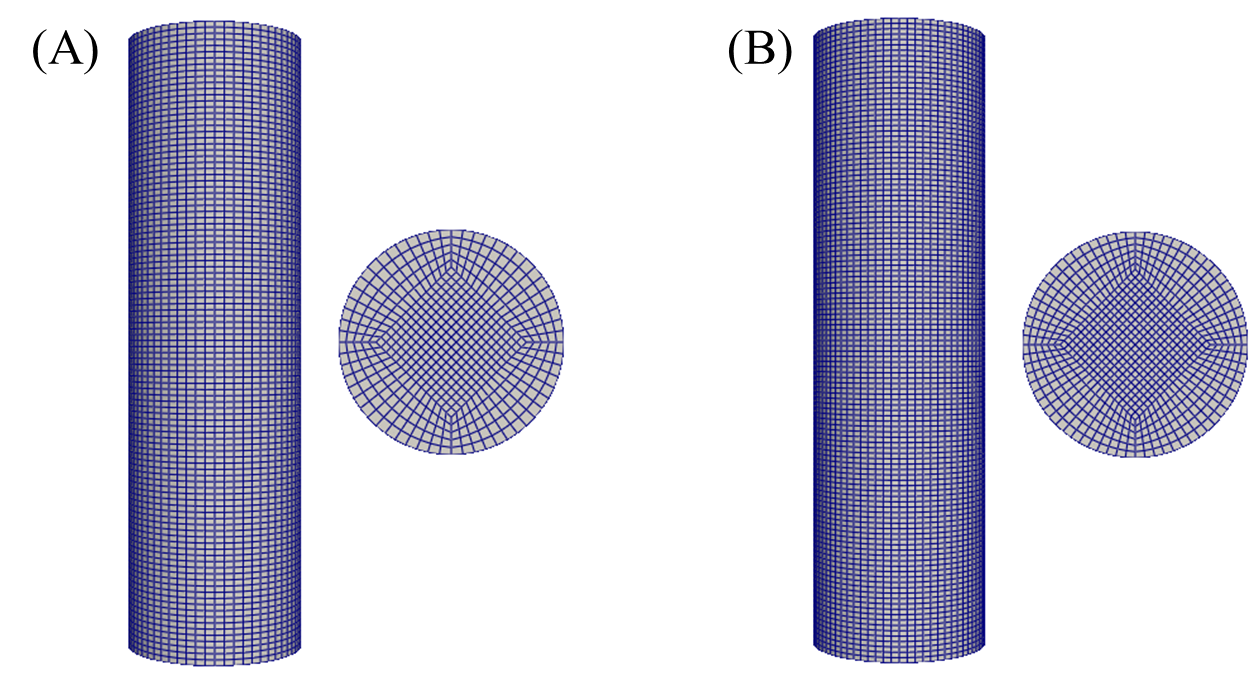


**Supplementary Figure 3.** Meshes used in CFD–DEM computation. **(A)** Original mesh; **(B)** Refined mesh.

The axial outlet gas velocity (at the central axis) changes against time as **Supplementary Figure 4**, showing no difference between the original and refined mesh. Thus, the original mesh density suffices for the dilute gas phase.


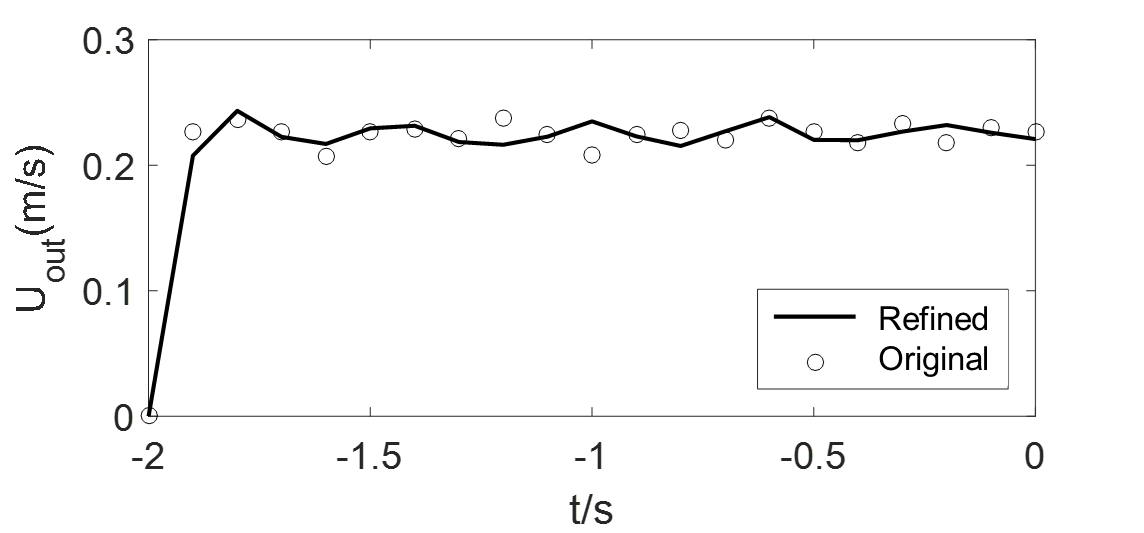


**Supplementary Figure 4.** Mesh refinement test of the axial outlet gas velocity. The negative time indicates the fluidization process before introducing reactive gases.

The overall solid conversion is plotted in **Supplementary Figure 5**. The refined result shows an equal initial rate to the original result; however, a discrepancy is observed in the middle stage (maximum 0.06). which is mainly induced by the error of volume fraction ($\alpha_{f}$). Given that the $\alpha_{f}$ of a cell undergoes a discontinuous change when a particle center crosses its face, greater errors are generated at a higher frequency for smaller cells. Moreover, the refined edge lengths are more close to a semi-particle-resolved method, but the fluid–particle interaction is still treated unresolved, instead of using an immersed boundary method. Thus, the grid size of unresolved CFD–DEM shall be set based on the particle diameter to reduce errors for the continuity equation.


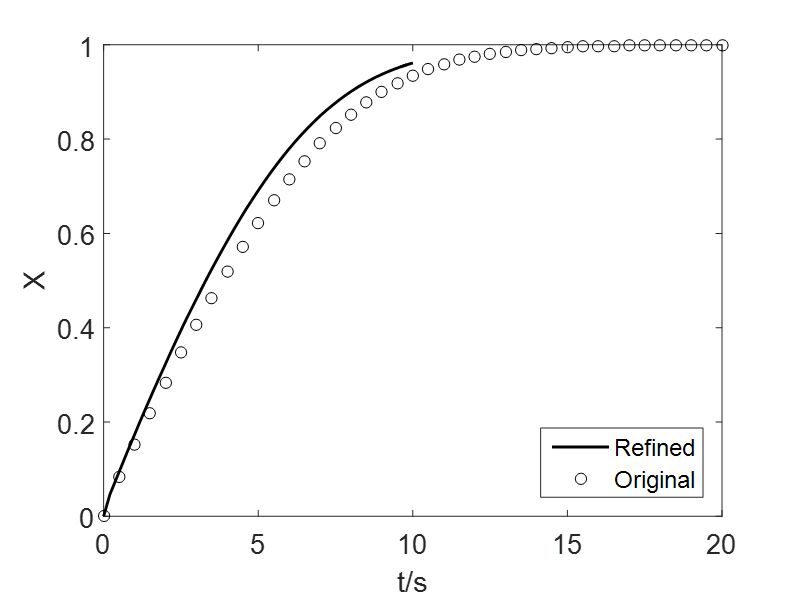


**Supplementary Figure 5.** Mesh refinement test of the overall solid conversion.

# References

Liu, L., Li, Z., Li, Z., Larring, Y., Li, Y., and Cai, N. (2021). Fast redox kinetics of a perovskite oxygen carrier measured using micro-fluidized bed thermogravimetric analysis. *Proc. Combust. Inst.* 38, 5259–5269. doi: 10.1016/j.proci.2020.06.160
